# Transcytosis via the late endocytic pathway as a cell morphogenetic mechanism

**DOI:** 10.15252/embj.2020105332

**Published:** 2020-07-13

**Authors:** Renjith Mathew, L Daniel Rios‐Barrera, Pedro Machado, Yannick Schwab, Maria Leptin

**Affiliations:** ^1^ Directors’ Research Unit European Molecular Biology Laboratory Heidelberg Germany; ^2^ Electron Microscopy Core Facility European Molecular Biology Laboratory Heidelberg Germany; ^3^ Institute of Genetics University of Cologne Cologne Germany; ^4^ Present address: National Institute for Science, Education and Research Bhubaneshwar India; ^5^ Present address: Institute for Biomedical Research Universidad Nacional Autónoma de México Mexico City Mexico; ^6^ Present address: Centre for Ultrastructural Imaging King's College London London UK

**Keywords:** cell polarity, *Drosophila*, endocytosis, membrane traffic, sorting, Development & Differentiation, Membrane & Intracellular Transport

## Abstract

Plasma membranes fulfil many physiological functions. In polarized cells, different membrane compartments take on specialized roles, each being allocated correct amounts of membrane. The *Drosophila* tracheal system, an established tubulogenesis model, contains branched terminal cells with subcellular tubes formed by apical plasma membrane invagination. We show that apical endocytosis and late endosome‐mediated trafficking are required for membrane allocation to the apical and basal membrane domains. Basal plasma membrane growth stops if endocytosis is blocked, whereas the apical membrane grows excessively. Plasma membrane is initially delivered apically and then continuously endocytosed, together with apical and basal cargo. We describe an organelle carrying markers of late endosomes and multivesicular bodies (MVBs) that is abolished by inhibiting endocytosis and which we suggest acts as transit station for membrane destined to be redistributed both apically and basally. This is based on the observation that disrupting MVB formation prevents growth of both compartments.

## Graphical abstract

Late endosomes act as hubs to regulate membrane material allocation during epithelial tube morphogenesis in the *Drosophila* tracheal system.

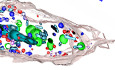

## Introduction

Most cells have specialized plasma membrane domains that serve dedicated physiological purposes. For instance, epithelial cells have an apical and a basal domain separated by adherens junctions and facing different parts of the body. Membrane and proteins are allocated to these domains in a way that is commensurate with their functions. For example, absorptive epithelia have massively enlarged apical domains organized in microvilli, and photoreceptor cells form specialized membranous outer segments for the light‐sensing rhodopsins. Errors in the proportions of membrane domains can have harmful consequences for organ function (Wodarz *et al*, 1995; Wilson, 2011; Zang *et al*, 2015). Therefore, the mechanisms that balance plasma membrane distribution are crucial for morphogenesis and tissue homeostasis.

Lipids are synthesized in the ER and trafficked to the plasma membrane via the Golgi apparatus or directly through ER–plasma membrane contact sites (Holthuis & Menon, 2014). Membrane delivery depends on fusion machinery, including SNAREs, and tethers such as the exocyst complex (Wu & Guo, 2015; Saheki & De Camilli, 2017). Due to technical limitations of direct labelling of membrane lipids *in vivo*, most studies addressing membrane trafficking follow cargo proteins. These are sorted generally at the trans‐Golgi network using receptors like components of the adaptor protein 1 (AP‐1) complex (Guo *et al*, 2014). Rab proteins can also participate in directing polarized secretion (Lerner *et al*, 2013; Bellec *et al*, 2018).

Material can also be passed from one domain to the other by transcytosis, which can occur either from apical to basal or vice versa, e.g., IgG and IgA in the gut (Fung *et al*, 2018). The main role described for transcytosis is to transport cargo from one side of an epithelium to the other. However, redistribution of plasma membrane may also be used for other purposes, including cell morphogenesis (Pelissier *et al*, 2003; Soulavie *et al*, 2018). The trafficking routes and the delivery mechanisms are not understood for these processes, nor is it known whether they are isolated special cases.

A cell type with sophisticated morphogenesis and pronounced specialization of membrane domains is the tracheal terminal cell in insects, which transports oxygen through a branched network of subcellular tubes. Tracheal terminal cells form long hollow branches, with the apical compartment forming the luminal membrane of each branch and the basal compartment facing the body's inner cavity. This architecture is formed by mechanisms shared by other lumen‐forming tissues like endothelial cells and Madin–Darby canine kidney (MDCK) cells grown in 3D (Sigurbjörnsdóttir *et al*, 2014). These mechanisms have been widely studied in *Drosophila* larval tracheal cells (Ghabrial *et al*, 2011; Baer *et al*, 2012; Schottenfeld‐Roames & Ghabrial, 2013; Jones *et al*, 2014; Rios‐Barrera *et al*, 2017), where they are, however, usually limited to endpoint phenotypes with only short‐term live imaging possibilities.

By contrast, cell morphogenesis and tube formation can easily be observed live in the embryo (Gervais & Casanova, 2010; JayaNandanan *et al*, 2014; Okenve‐Ramos & Llimargas, 2014; Ricolo *et al*, 2016), where the first branch forms. The subcellular tube develops by an invagination of the apical compartment, and membrane is added throughout the length of the invaginating tracheal tube (Gervais & Casanova, 2010). The tube grows in unison with the basal domain of the cell, and as the branch and its tube extend, trafficking markers are seen throughout the cell, often associated with the tube (Gervais & Casanova, 2010; JayaNandanan *et al*, 2014; Schottenfeld‐Roames *et al*, 2014). Studies in larvae show that failures in endocytosis, exocytosis and secretion can all affect the growth of both membrane domains as judged by reduced cell branching (Jones *et al*, 2014; Schottenfeld‐Roames *et al*, 2014; preprint: Rios‐Barrera *et al*, 2017). However, only the loss of endocytic components has been associated with defects in subcellular tube size and architecture (Schottenfeld‐Roames *et al*, 2014). How these vignettes of knowledge fit into a picture of an integrated membrane delivery mechanism that balances apical and basal delivery is not known. Here, we address this by perturbing membrane dynamics and looking at the redistribution of membrane and protein markers during terminal cell development in the embryo.

## Results

### Organization of membrane domains during subcellular tube morphogenesis

**Figure 1:**
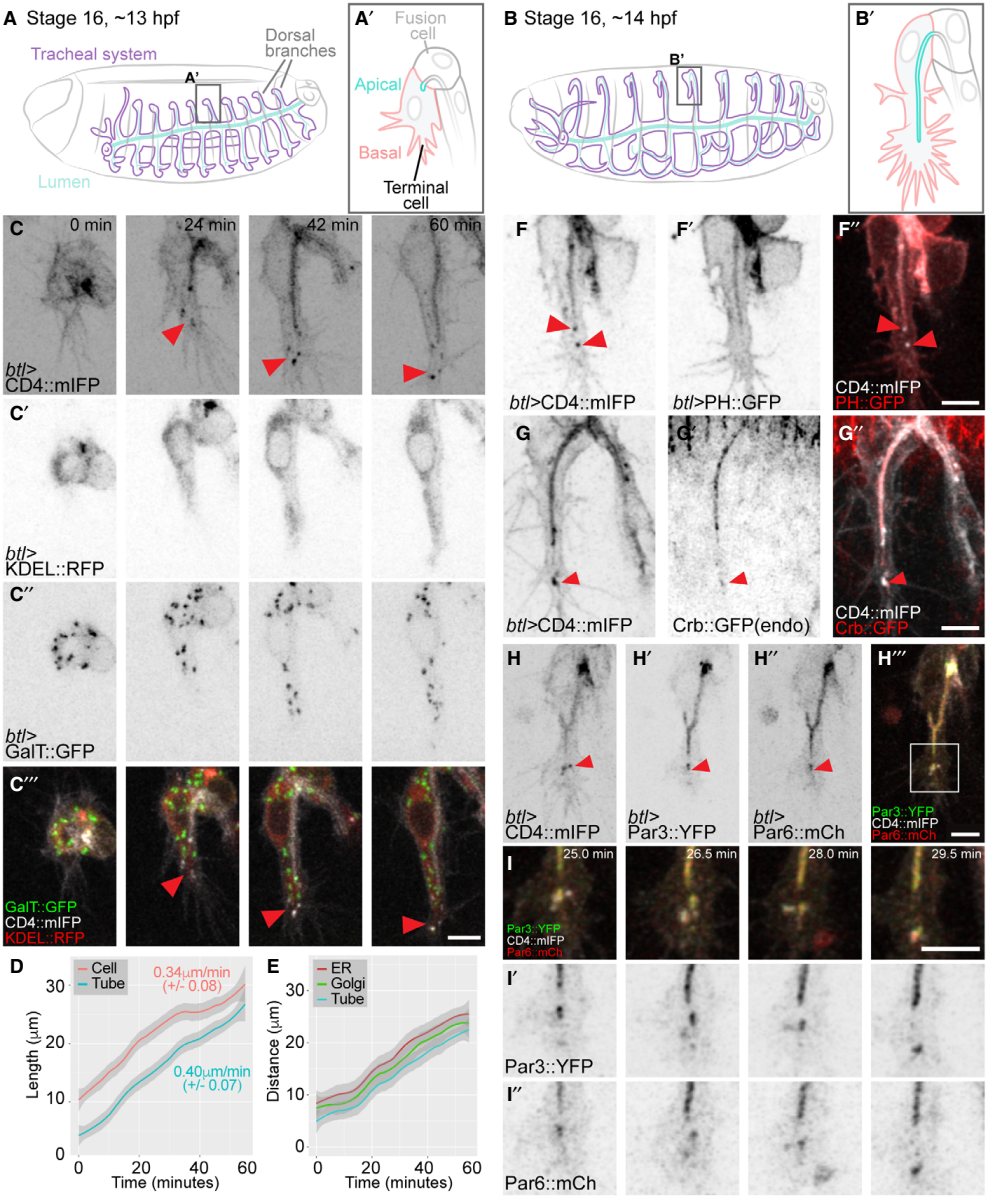
Membrane organization during tube morphogenesis A, BDiagram of the organization of the tracheal system at the onset of terminal cell branching (A) and 1 h later (B).C–C‴Terminal cell expressing reporters for membrane (C), ER (C′) and Golgi apparatus (C″).DMean and 95% confidence interval of tube and branch length during the first hour of growth. Numbers represent the rate of growth estimated from the slope of the curve ± SD, *P* = 0.39, Mann–Whitney U‐test, *n* = 6.EDistance of the tip of the tube, and the leading ER and Golgi accumulations from the cell junction that defines the base of the cell. Line and shading represent mean and 95% confidence interval, *n* = 4.F–ITerminal cells expressing the general membrane marker CD4::mIFP in combination with different membrane or polarity markers: PH::GFP, a PIP_2_ sensor commonly used as apical plasma membrane marker (F′), an endogenously GFP‐tagged Crb (G′), polarity proteins Par3::YFP (H′) and Par6::mCherry (H″). The boxed region in (H‴) is shown at higher magnification in (I) over 4 time points.Data information: Red arrowheads: CD4::mIFP vesicles and associated markers at the tip of the cell. Anterior is left, and dorsal is up. Scale bars: 5 μm.Source data are available online for this figure.

Tracheal terminal cells elongate with their subcellular, apical tube and outer, basal membrane compartment growing at the same rate (Fig 1A–D). Since lipids are produced in the ER, and membrane proteins and secreted components of the tube must pass through the Golgi, these organelles are likely critical for the expansion of the plasma membrane. To study their distribution, we used KDEL::RFP as a maker for ER and the Golgi‐targeting sequence of β‐1,4‐galactosyltransferase fused to GFP (GalT::GFP) as a trans‐Golgi marker, together with CD4::mIFP, a membrane reporter (Yu *et al*, 2015) that is enriched in the plasma membrane but also seen in other subcellular membrane compartments. Both organelles are present in the cell body, throughout the length of the growing branch and in the growth cone ahead of the tube (Fig 1C and E).

We also noticed accumulations of CD4::mIFP‐labelled membrane material in the space between the extending filopodia of the growth cone and the tip of the growing tube (Fig 1C). A similar accumulation in this position has been reported for Par3 (which is mainly associated with the apical membrane) but suspected to be an artefact of Par3 overexpression (Gervais & Casanova, 2010). We nevertheless considered the possibility that these structures might be part of the extending tube, or perhaps nascent plasma membrane, and analysed them with a range of membrane markers. They were also seen with other general membrane reporters (Appendix Fig S1A–C), but not with markers considered to be selective for the plasma membrane, such as the Pleckstrin homology domain (PH) of PLCδ fused to GFP or mCherry. These labelled only the tube and the basal membrane of the cell (Fig 1F; Appendix Fig S1B and C; Movie EV1). This suggests that the material does not correspond to plasma membrane.

The presence of Par3 in this region is therefore intriguing, and we tested other characteristic apical membrane‐associated proteins for their distribution. A GFP inserted into the *crumbs* (*crb*) locus, Crb::GFP, was seen in its normal location at the tube membrane and also with the CD4 vesicles near the tip of the cell (Fig 1G), similar to an overexpressed construct (Appendix Fig S1D). Par3 and Par6 also localized to CD4 vesicles ahead of the tube (Fig 1H). On occasions where different polarity markers were associated with the same vesicle, their overlaps remained partial (Fig 1I). The fact that Crb::GFP fluoresces indicates that it is not recently synthesized [GFP maturation time is more than 1 h (Balleza *et al*, 2018)] and that this compartment therefore does not represent an intermediate along the biosynthetic pathway from the Golgi to the plasma membrane. Instead, we conclude that most likely these structures are endosomes that arise from the apical membrane. This conclusion was supported by high time resolution imaging of Par3 and CD4. The Par3 ahead of the tube appeared to originate from the tube and move to the tip (Appendix Fig S1E; Movie EV2), as previously reported (Gervais & Casanova, 2010).

**Figure EV1:**
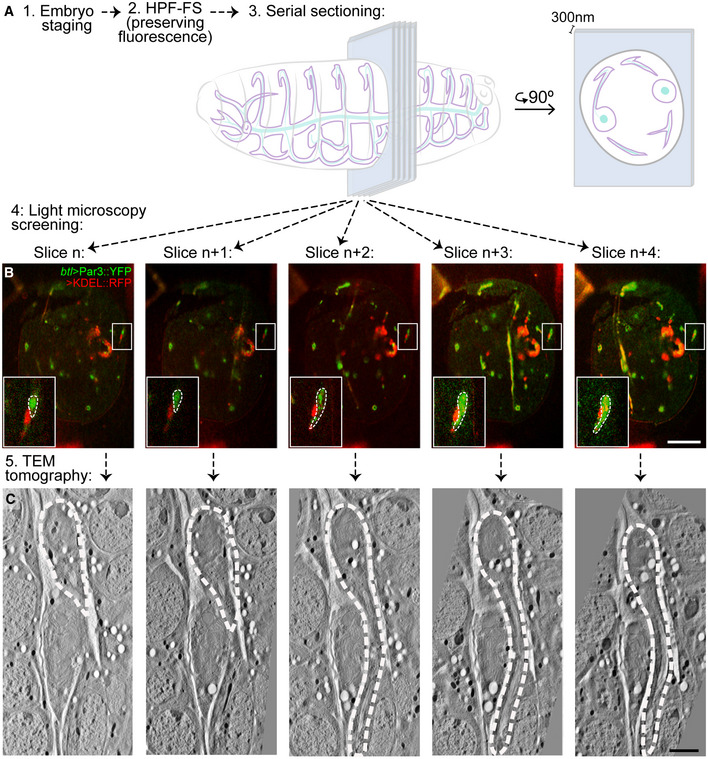
Correlative light and electron microscopy workflow to identify terminal cells Embryos were processed for EM while preserving the fluorescence signal, and then sectioned at 300 nm.Physical sections (slices) were then analysed by fluorescence microscopy, and once a terminal cell was identified (Slice *n*), the adjacent sections were collected to recover the complete cell (Slice *n* to *n*+4).The recovered slices were then imaged by electron tomography and digitally aligned.Data information: Scale bars: 20 μm (B); 2 μm (C). White dashed line in (B and C) shows the outline of the terminal cell.

To understand the nature of this domain, we used serial section electron tomography. To generate an atlas of organelle distribution throughout the length of the cell, we initially screened serial sections manually to identify terminal cells. Though feasible, this labour‐intensive workflow allowed only limited analyses. To avoid screening using EM, we turned to correlative light and electron microscopy (CLEM), building on previous protocols that preserve the signal from fluorescent proteins (Nixon *et al*, 2009; Kukulski *et al*, 2011). Embryos expressing KDEL::RFP and Par3::YFP under *btl‐gal4* were fixed and serially sectioned to cover at least one full embryonic segment (200 sections of 300 nm). The fluorescent signal allowed rapid identification of the terminal cells to be imaged by high‐resolution electron tomography (Fig EV1).

**Figure 2:**
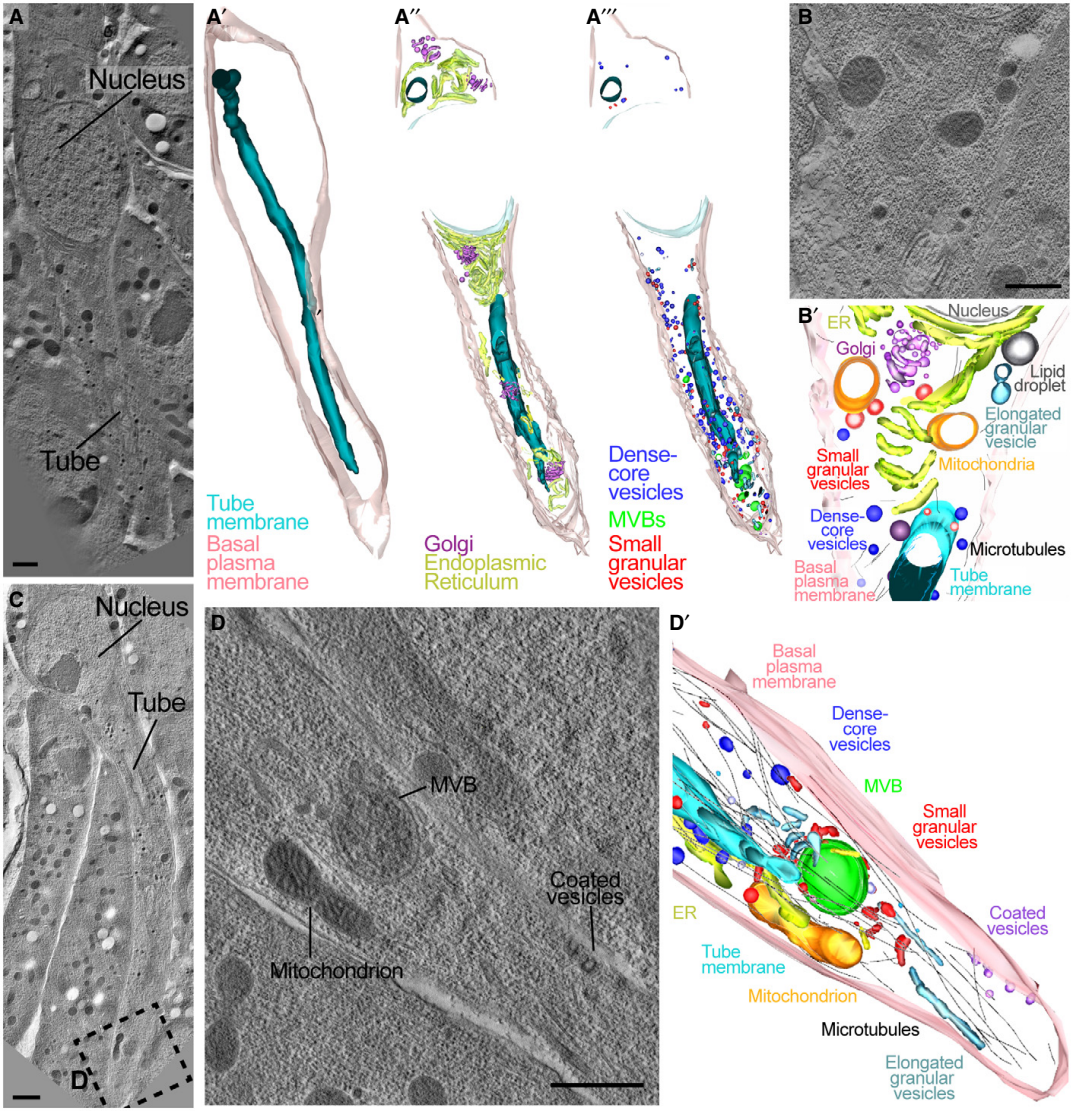
Reconstruction of organelles in a terminal tracheal cell by electron tomography A–DTEM tomograms from high‐pressure frozen embryos.A–A′Low magnification (2.5 nm voxels) tomogram and 3D reconstruction from 10 serial 300‐nm sections that cover one terminal cell.A″–A‴3D reconstructions derived from higher‐resolution tomograms showing different organelles in the cell.B–B′Example of an individual high‐resolution (0.78 nm voxels) tomograms used to generate 3D reconstructions.CLow‐resolution tomogram of another cell; the dotted box is magnified in (D).DHigh‐resolution tomogram.D′Model derived from 4 serial 3D reconstructions including the one shown in (D).Data information: Scale bars: 1 μm (A, C); 500 nm (B, D).

**Figure 3:**
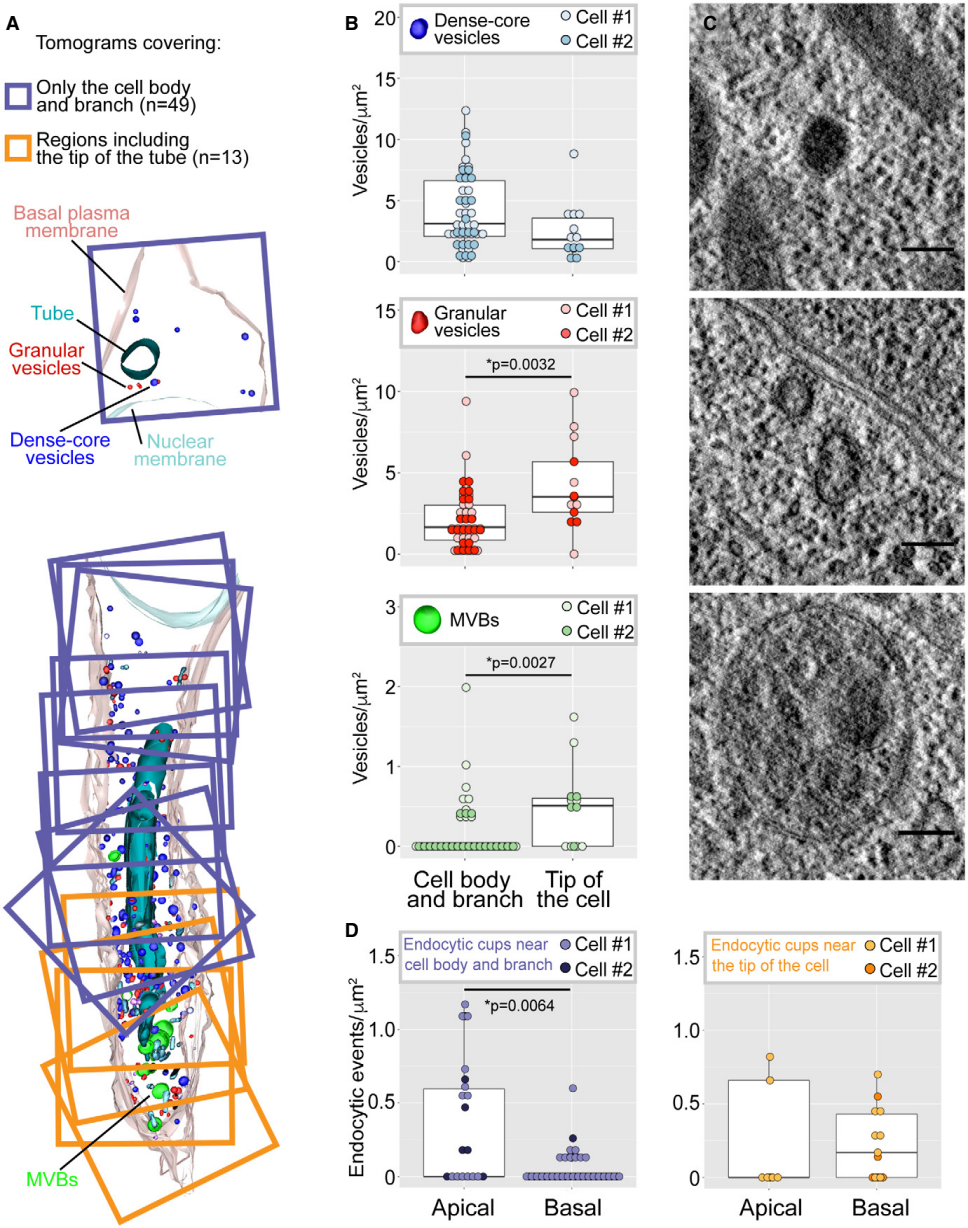
Representative vesicle types and their distribution in the terminal cell Cell model composed of superimposed 3D reconstructions of high‐resolution TEM tomograms covering different regions of the cell. Each square is one 3.2 × 3.2 × 0.17 μm 3D reconstruction. The tomograms were assigned to two categories for quantitative evaluation: those including the tip of the tube (orange) and the rest (purple).Distribution of three commonly seen classes of vesicles, expressed as their density per area of cytoplasm screened, in the two regions of the cell. Box plots represent median, interquartile range (IQR) and IQR*1.5 below and above the IQR. *n* = 62 tomograms from two different cells.Representative examples for each vesicle class are shown next to the quantification plots.Distribution of endocytic events at the apical or basal membrane compartment, expressed as density per membrane surface screened. Left shows tomograms corresponding to the cell body and branch, and right shows tomograms corresponding to the tip of the cell. Box plots represent median, interquartile range (IQR) and IQR*1.5 below and above the IQR. *n* = 48 tomograms from two different cells.Data information: Significance was determined using Mann–Whitney U‐test. Scale bars: 100 nm.Source data are available online for this figure.

We first obtained low‐resolution overviews of cell morphology (Fig 2A and C) and then used higher‐resolution tomograms to manually trace organelles (Fig 2B and D). The tomograms confirmed the distribution of ER and Golgi we had observed by light microscopy, with both organelles spread throughout the length of the cell (Fig 2A–A″). We also found a range of vesicles with distinctive electron densities, sizes and distributions (Fig 3A–C). Many MVBs and large electron‐dense vesicles were seen close to the tip of the tube (Fig 3A and B; Movie EV3). This, together with the presence of Crb, Par3 and Par6 in this location, suggests extensive membrane trafficking or active recycling events throughout the cell and particularly in the growing tip.

### The role of endocytosis in terminal cell morphogenesis

The basal plasma membrane and the subcellular tube grow in unison. Hence, there must be a mechanism to balance the delivery of membrane between the two domains. There are in principle two ways to achieve this: (i) membrane is delivered from the ER and Golgi directly and in the correct measure to each compartment. (ii) ER and Golgi deliver membrane to one compartment, and part of this material is then retrieved and transported towards the other via transcytosis. These models can be distinguished experimentally, because the latter requires endocytosis from the plasma membrane for the shuttling. To test this, we blocked endocytosis using a temperature‐sensitive allele of *dynamin*,* shibire* ^*ts*^ (Koenig & Ikeda, 1989), which can be inactivated within 15 min by shifting the embryos to 34°C.

**Figure 4:**
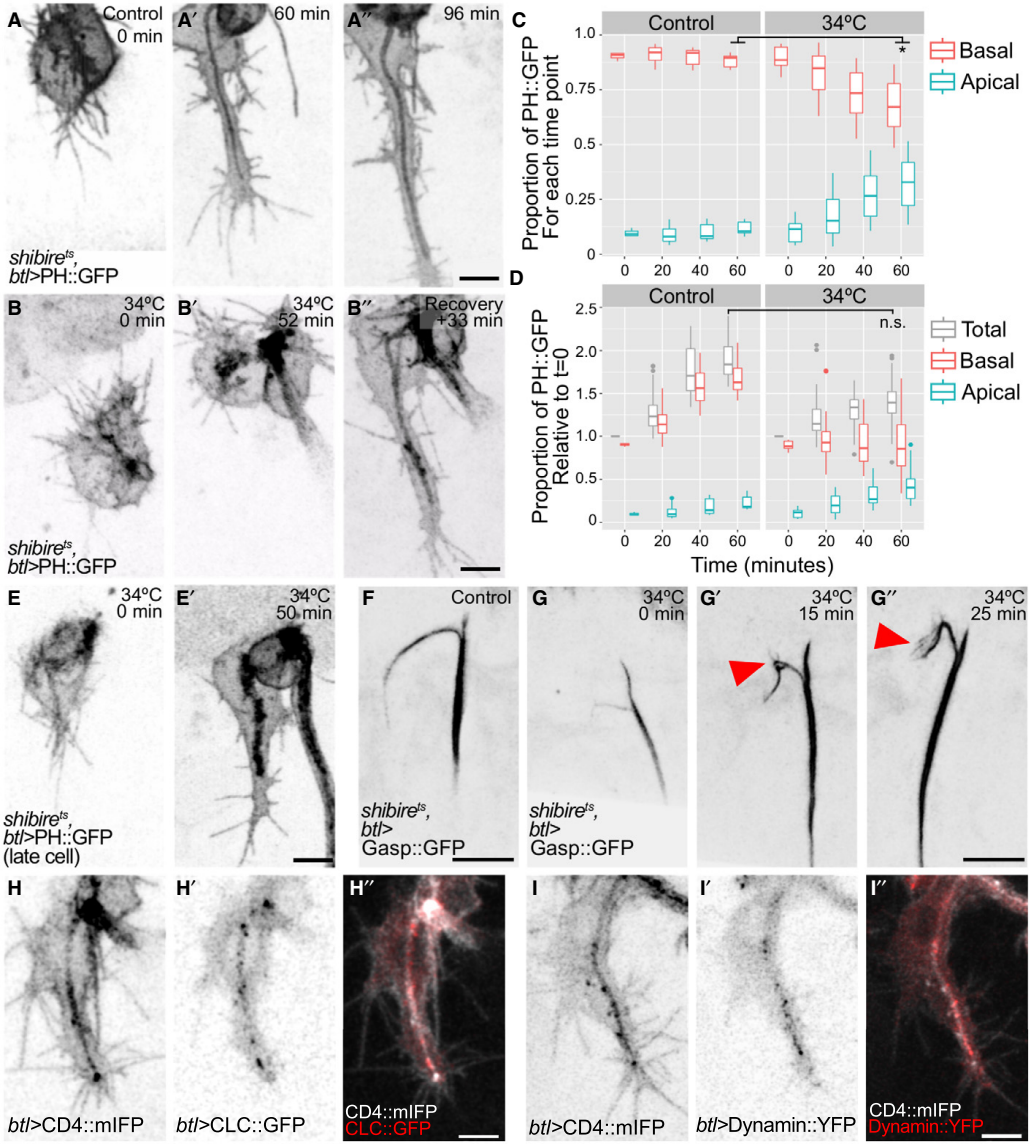
The role of endocytosis in terminal cell growth A–EDistribution of the plasma membrane reporter PH::GFP in control cells (A–A″) and in cells where dynamin activity had been blocked using a temperature‐sensitive allele of *dynamin* (*shibire* ^*ts*^, B, E). In (B), it was inactivated at the onset of tube formation, whereas in (E), it was inactivated ˜15 min after the tube had begun to form. (C–D) Distribution of PH::GFP fluorescence intensity in control and *shibire* ^*ts*^ cells. Data from 1‐ to 2‐min interval time lapses were collected in windows of 20 min each (except for t = 0). Box plots represent median, interquartile range (IQR) and IQR*1.5 below and above the IQR. (C) Proportion of signal in the apical and in the basal membrane compartment over time in control cells (*n* = 4) and also in cells where dynamin was inactivated (*n* = 5), *P = 0.015, Mann–Whitney U‐test. (D) Total fluorescence intensity of PH::GFP over time in controls (*n* = 4) and in cells where dynamin was inactivated (*n* = 5), n.s.: *P* = 0.063, Mann–Whitney U‐test.F, GDistribution of the luminal reporter Gasp::GFP in control cells (F) and in cells where dynamin was inactivated (G). Arrowheads point to protrusions sprouting from the lumen of the terminal cell.H, IDistribution of the general plasma membrane marker CD4::mIFP in combination with the clathrin light chain fused to GFP (CLC::GFP, H) and with Dynamin::YFP (I).Data information: Scale bars: 5 μm. Source data are available online for this figure.

We blocked dynamin at the onset of tube formation in cells expressing PH::GFP, a construct commonly used as a marker for apical membrane but which is also visible in the basal plasma membrane (Fig 4A and B). Unlike control cells, where basal and apical membrane domains expanded at similar rates (Fig 4A, Movie EV4), cells in which dynamin was inactivated failed to grow properly. *shibire* ^*ts*^ cells showed an excessive increase in membrane material inside the cell whereas the basal membrane failed to grow (Fig 4B′), leading to a shift in the proportions of membrane on each domain. In control cells, the proportion of fluorescent material in each compartment remains constant during cell growth (12% in the apical versus 88% in the basal domain, ± 2 SD), whereas it gradually increased in *shibire* ^*ts*^ cells, reaching up to 35% in the apical and 65% in the basal ± 10 SD (Fig 4C). Blocking dynamin function in older cells where the basal membrane and the tube had already extended led to the accumulation of the marker throughout the length of the tube (Fig 4E, Movie EV4). The defects in cell and tube growth were reversible: shifting the embryos back to the permissive temperature restored the expansion of the basal membrane and resulted in partial or complete resolution of the membrane accumulation in the tube domain (Fig 4B″, Movie EV4).

While it was evident that the outer membrane domain expansion was strongly reduced after blocking endocytosis, it was less clear whether the internal membrane pool corresponded to the normal amount that had simply been compacted within a smaller volume, or whether more apical membrane was present. The finding that total fluorescence intensity (basal plus apical) of the cell increased at the normal rate after dynamin inactivation (Fig 4D) shows that plasma membrane synthesis and delivery *per se* were not affected. Our measurements indicate that upon dynamin inactivation, a similar amount of membrane material as would normally have been added to the basal domain had instead accumulated in the subcellular tube, in addition to the material that is normally delivered there.

We confirmed the identity of the material within the cell as apical membrane by the presence of Crb and of Gasp::GFP, a protein secreted into the tracheal lumen. Crb colocalized with the membrane reporter within the cell (Appendix Fig S2A, B, D and E), and it remained associated with the tube after recovery (Appendix Fig S2C and F). Gasp::GFP formed complex ramifications that sprout from the subcellular tube (Fig 4F and G). These results show that dynamin, and therefore most likely endocytosis, is needed for the correct allocation of the appropriate amounts of membrane to the basal and apical compartments. Specifically, membrane is delivered to the apical domain, and it remains there in the absence of endocytosis, while insufficient or no membrane finds its way to the basal domain. According to this model, endocytosis should be more prominent at the apical domain. Consistent with this, both the clathrin light chain (CLC) and dynamin itself were present throughout the cell but they were more abundant around the tube than in the basal membrane (Fig 4H and I). This was seen in EM images, where we found a higher density of endocytic events at the apical than at the basal membrane (Fig 3D).

**Figure EV2:**
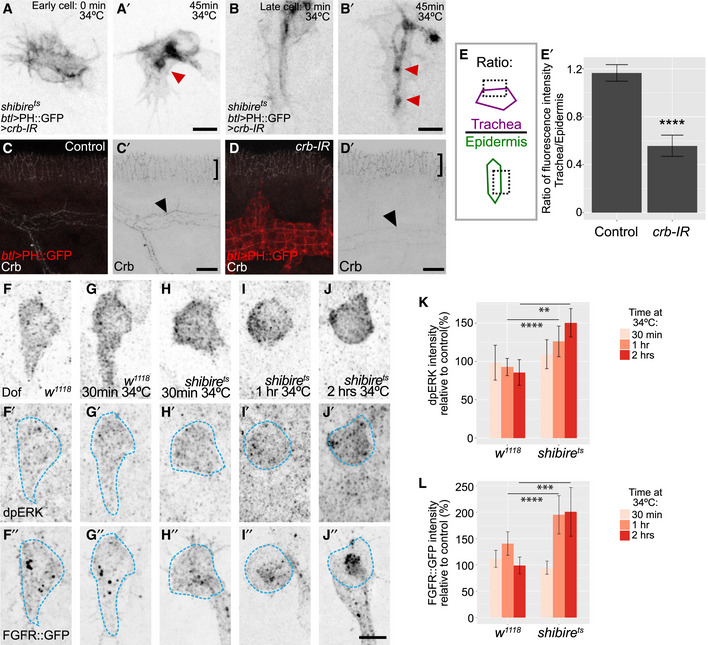
Effect of blocking endocytosis on Crb and FGF signalling A, BEffect of Crb interference RNA (Crb‐IR) on *shibire* ^*ts*^ terminal cells. Red arrowheads point to sites of membrane accumulation in the apical compartment. (A) Cell at the onset of tube formation; (A) at the beginning of dynamin inactivation and (A′) after 45 min. (B) Later‐stage terminal cell; (B) at the beginning of dynamin inactivation and (B′) after 45 min.C, DFixed embryos stained for Crb. Black arrowheads point to signal in the trachea and squared brackets show signal in the epidermis, which was used for normalization. (C) Control embryos (siblings without driver or UAS constructs). (D) Embryos expressing PH::GFP and *crb‐IR* under *btl‐gal4*.E–E′Quantification of Crb fluorescence intensity for 4 control and 5 *crb‐IR* embryos. Data are plotted as ratio of signal in tracheal cells compared to epidermal cells,  ± SD; ****P > 0.0001, two‐tailed *t* test.F–JFixed embryos stained for Dof as a terminal cell marker, dpERK (F′‐J′, terminal cell position is highlighted in blue) and FGFR::GFP from the fTRG collection (F″‐J″).K–LQuantification of dpERK and FGFR::GFP fluorescence intensity. Data are plotted as per cent relative to control (*w* ^*1118*^ *,* for each time point), ± SD. We analysed two cells per embryo; number of embryos analysed: *w*, control (for 30 min at 34°C), *n* = 13; *w*, 30 min at 34°C, *n* = 7; *shi*, control (for 30 min at 34°C), *n* = 2; *shi*, 30 min at 34°C, *n* = 11; *w*, control (for 1 h at 34°C), *n* = 18; *w*, 1 h at 34°C, *n* = 11; *shi*, control (for 1 hr at 34°C), *n* = 4; *shi*, 1 h at 34°C, *n* = 9, *w*, 2 h at 34°C k, *n* = 5; *shi,* control (for 2 h at 34°C), *n* = 6; *shi*, 2 h at 34°C, *n* = 6. **P = 0.009, ***P = 0.0002, ****P > 0.0001, Kruskal–Wallis test and Dunn's test for multiple comparisons. Data information: Scale bars: 5 μm. Source data are available online for this figure.

To test whether raised levels of Crb were responsible for the excessive apical membrane, as reported in other contexts (Wodarz *et al*, 1995; Pellikka *et al*, 2002; Schottenfeld‐Roames *et al*, 2014), we knocked down *crb* (Fig EV2A and B). This approach reduced the level of Crb to ~50% of that seen in control cells (Fig EV2C–E). However, this reduction did not alleviate the accumulation of apical membrane. We conclude that blocked endocytosis directly interferes with bulk apical membrane retrieval.

We also considered other potential reasons for the lack of basal membrane growth. Because endocytosis is involved in receptor tyrosine kinase signalling (Villaseñor *et al*, 2016) and FGF signalling is required for terminal cell growth, the growth defect could be due to impaired FGF signalling. To test this, we quantified ERK phosphorylation, a signature of FGF receptor activation (Gabay *et al*, 1997). Blocking dynamin led to a slight increase in di‐phospho‐ERK. This was not simply due to the temperature increase, as it was not observed in control embryos shifted to 34°C (Fig EV2F–K). Thus, blocking dynamin function does not prevent FGF signalling activation, and if anything, it results in a slight increase in the activation of ERK.

Having excluded other explanations, we suggest that the lack of basal membrane growth and overgrowth of tube membrane are functionally connected and that the normal balance between the two requires endocytosis. The simplest scenario would be transport of apical membrane material to the basal domain by transcytosis.

### Membrane morphology and distribution in control and *shibire* ^*ts*^ cells

**Figure 5:**
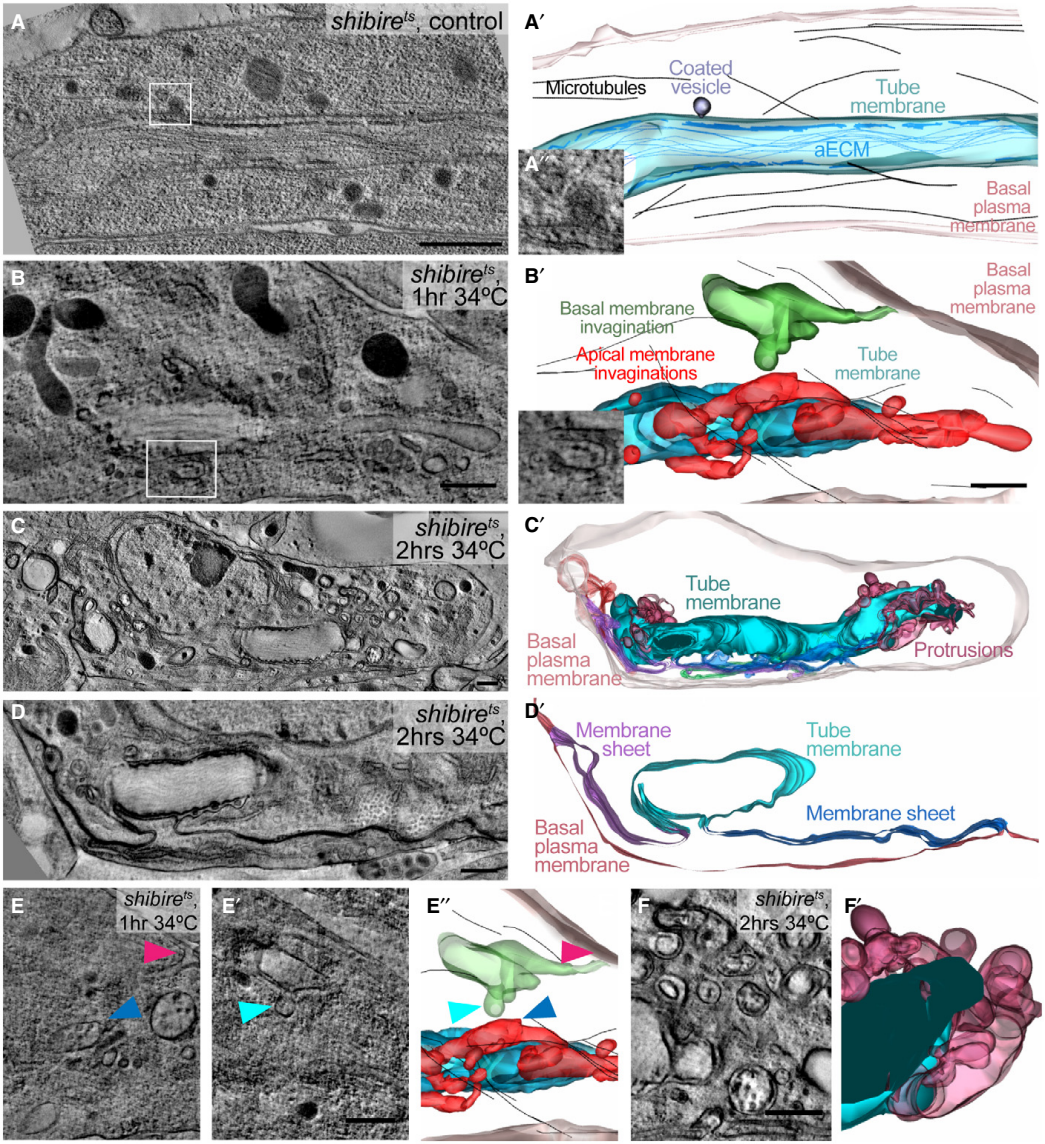
Effects of dynamin inactivation on membrane morphology A–FTEM tomograms and 3D reconstructions of terminal cells in *shibire* ^*ts*^ embryos. (A) Control. (B‐F) Kept at 34°C for 1 (B, E) or for 2 h (C, D, F). Boxed regions in (A) and (B) are magnified in (A″) and (B″). (A′, B′) Reconstructed 3D models. The tube membrane (cyan) is characterized by the electron‐dense apical extracellular matrix (aECM). Invaginations with connections that can be traced to the tube membrane are red, and invaginations from the basal plasma membrane are green. The basal membrane is light pink. (C‐D′) *shibire* ^*ts*^ terminal cell after 2 h at 34°C. (C′) 3D reconstruction, colour coding for basal and tube membrane is the same as above. Membrane that is continuous with both the apical and the basal membrane is shown in green, purple and dark blue. (D‐D′) Higher magnification of a region in the same cell at a level where membrane sheets (purple and dark blue) bridge the apical and basal plasma membrane domains. (E‐E″) Two focal planes and model of a tomogram from the cell shown in (B) where a basal (pink arrowhead) and an apical invagination (blue) are seen in close proximity (closest distance is marked by cyan and blue arrowheads). (F‐F′) Apical membrane overgrowth regions of the cell shown in (C). The genotype of the embryos was *shibire* ^*ts*^ *; btl>*KDEL::RFP, Par3::YFP. The cell shown in (C–D) was found and acquired without the CLEM approach.Data information: Scale bars: 500 nm.

**Figure EV3:**
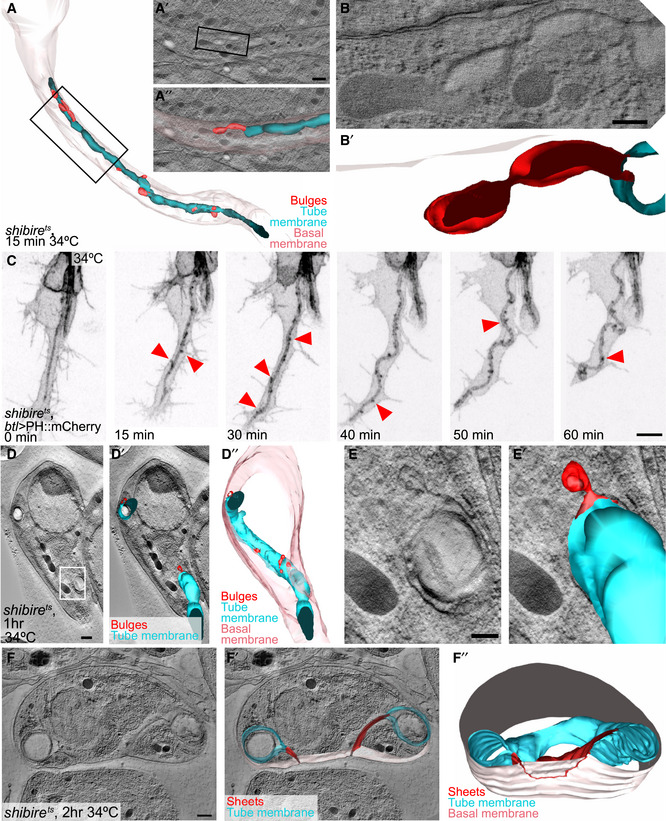
Effects of dynamin inactivation on membrane morphology A, BOlder *shibire* ^*ts*^ terminal cell that had already formed a long branch and tube before dynamin inactivation, fixed 15 min after inactivation. (A) Reconstruction of the entire cell. (A′, A″, B, B′) Higher magnification details of the cell, tomograms and reconstructions.C
*shibire* ^*ts*^ terminal cell expressing PH::mCherry. Red arrowheads point to puncta of fluorescent material at the tube membrane.D–FTEM tomograms and 3D reconstructions of older *shibire* ^*ts*^ terminal cells similar to (A), but after 1 h (D–E) and 2 h (F) at restrictive temperature. Box in (D) is magnified in (E). (F″) The position at which the sheet between apical and basal is connected to the basal membrane is traced in red on the outside view of the basal membrane. The cells shown in (D–F) were found and acquired without the CLEM approach.Data information: Scale bars: 500 nm (A, D, F), 200 nm (B, E), 5 μm (C).

To see the effect of blocking dynamin on the membrane or other compartments within the cell, we used electron tomography. Control cells had smooth tube membranes, with apical extracellular matrix (aECM) in the lumen of older cells. This appeared as long fibres curling inside the tube and as electron‐dense depositions adjacent to the plasma membrane (Fig 5A). In cells fixed after 15 min of dynamin inactivation, the tube membrane appeared largely similar to the control (Fig EV3A and B), consistent with the minor effects on cell morphology seen in live observations (Fig EV3C). However, we also found bulges protruding from it into the cytosol (Fig EV3B). These were also visible after 1 h of dynamin inactivation, where additionally the tube membrane appeared highly irregular (Figs 5B and EV3D and E, Movie EV5). We interpret these irregularities as endocytic events in which scission from the membrane had failed to occur, and indeed in several instances, these structures were surrounded by particulate electron‐dense structures resembling clathrin coats (Fig 5B″).

After 2 h of dynamin inactivation, the morphology of the tube membrane was severely affected. The cells contained complex ramifications of the tube membrane together with its aECM (Fig 5C and F), which resembled the structures we had seen by light microscopy using Gasp::GFP (Fig 4G). The cells also showed more dramatic defects. Some invaginations from the tube membrane could be traced all the way to the basal plasma membrane, with which they were clearly contiguous (Figs 5D and EV3F, Movie EV5). All cells had instances of large sheets of membrane connecting different parts of the tube and the plasma membrane. These sheets did not represent auto‐cellular junctions as seen in other tracheal cells (Francis & Ghabrial, 2015) since they contained neither Ecad nor FasIII (Appendix Fig S2G–J). In several places, the space between the two apposed plasma membrane sheets contained material resembling the content of MVBs (Figs 5B and E, and EV3E). If these sheets are the result of fusion events between invaginations emanating from the apical and basal plasma membrane, then these events must happen rarely and, when they do happen, rapidly, since we found no clear intermediates in our tomograms, such as apical invaginations reaching to the basal plasma membrane. However, we saw several instances of long invaginations from the apical membrane and occasional ones from the basal membrane, and in one cell, the surfaces of two such invaginations came within 500 nm of each other (Fig 5E, Movie EV5).

### Distribution of basal cargo in control and *shibire* cells

If the basal compartment is derived from the apical compartment by endocytosis followed by transcytosis, then general secretion from the ER/Golgi should be directed predominantly towards the apical compartment. This should also apply for transmembrane proteins that are destined for the basal membrane. If secretion follows the route we postulate, blocking dynamin should lead to basal cargo accumulating in the apical compartment before it can reach the basal domain. Hence, we looked at the transport of two known basal transmembrane proteins expressed in tracheal cells, the FGF receptor (FGFR) Breathless and the integrin β‐chain Myospheroid (βPS‐integrin).

**Figure 6:**
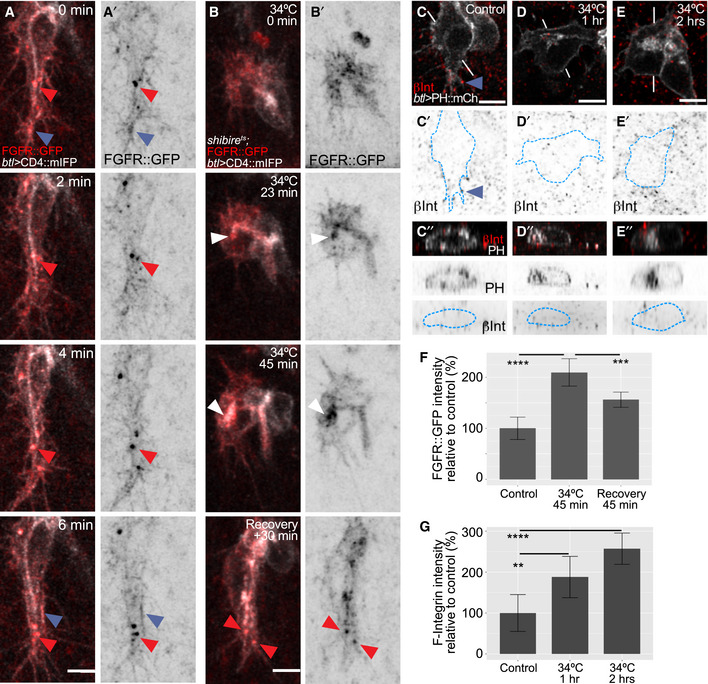
Effect of dynamin inactivation on the distribution of basal proteins A, BTerminal cells expressing the membrane marker CD4::mIFP under *btl‐gal4* and FGFR::GFP under its own promoter (from the fTRG library). (A) Time lapse imaging of a control cell. Blue arrowheads point to filopodia and basal plasma membrane, and red ones point to puncta containing CD4::mIFP and FGFR::GFP. (B) *shibire* ^*ts*^ cell imaged before dynamin inactivation, after 23 and 45 min of inactivation, and after 30 min of recovery. White arrowheads point to FGFR::GFP accumulation at the apical compartment.C–ESingle confocal planes of terminal cells expressing PH::mCherry and stained for βPS‐integrin. The outline of the cells was traced using the PH::mCherry signal and is shown as a blue dashed line. (C) Control. Blue arrowheads: βPS‐integrin signal in filopodia. (D) 1 h at 34°C; (E) 2 h at 34°C. (C″–E″) Orthogonal views of the lines shown in white in (C–E).F, GQuantification of fluorescence intensity from stained embryos of FGFR::GFP (F) and of βPS‐integrin (G). Data are plotted as mean ± SD; significance was assessed using one‐way ANOVA with Dunnett's correction for multiple comparisons. **P = 0.0022, ***P = 0.0003, ****P < 0.0001. Number of cells analysed for (F): control, *n* = 8; 1 h at 34°C, *n* = 6; recovery, *n* = 8. (G). For (G): control, *n* = 7; 1 h at 34°C, *n* = 8; 2 h at 34°C, *n* = 8.Data information: Scale bars: 5 μm.Source data are available online for this figure.

In control cells, both proteins mostly localized to the basal filopodia, although FGFR was also seen in large puncta near the tip of the cell (Fig 6A and C). In *shibire* ^*ts*^ embryos, both were abnormally accumulated at the apical membrane (Figs 6B and D–G, and EV2F″–J″ and L).

These findings support our conclusion that the initial delivery of membrane from the Golgi goes mainly to the apical domain and fails to be redistributed basally in the absence of endocytosis. An alternative interpretation for the apical mislocalization would be that the defect in *shibire* ^*ts*^ cells was not one of the failed retrievals of basal proteins, but that cargo was misdirected immediately after the Golgi upon dynamin inactivation, as observed in a mammalian model (Deborde *et al*, 2008). If this is the explanation for the abnormal localization of βPS‐integrin and FGFR, restoring dynamin function should not restore their proper localization. Conversely, if FGFR and βPS‐integrin mislocalization was the result of failed membrane endocytosis at the tube, restoring endocytosis should correct their faulty localization. We observed by live imaging (Fig 6B, Movie EV6) and also by immunostainings (Fig 6F, Appendix Fig S2K–M) that after restoring dynamin function, FGFR distribution to the basal membrane was re‐established (Fig 6B and F; Appendix Fig S2M; Movie EV6). These results support the model where basal proteins are delivered to the apical membrane first and are then transcytosed to the basal membrane by dynamin‐mediated endocytosis.

### Vesicle trafficking and conversion

**Figure 7:**
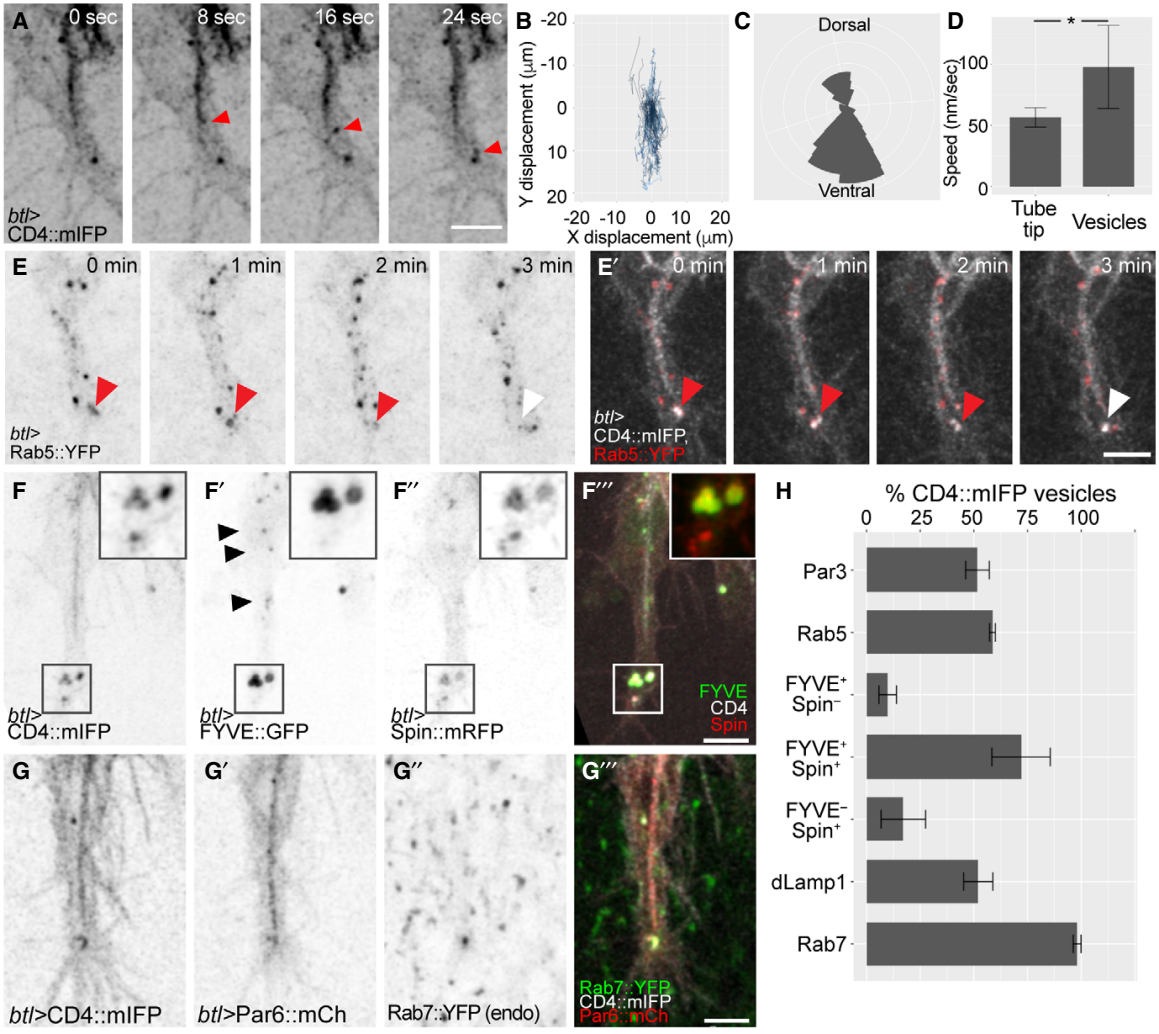
Composition and distribution of endosomal compartments during terminal cell growth A–DHigh time resolution imaging of the membrane marker CD4::mIFP expressed under *btl‐gal4*. (A) Example of a large CD4 vesicle (arrowhead) rapidly moving towards the tip of the terminal cell. (B–D) Analysis of 28 CD4 vesicles in five terminal cells. (B) Trajectories of CD4 vesicles with their original position mapped to the origin of the plot. (C) Rose diagram of the trajectories shown in (B). (D) Speed of CD4 vesicles compared to speed of tube growth measured over 25 min. Data are plotted as mean ± SD. *P < 0.001, Mann–Whitney U‐test.E–HTerminal cells expressing the membrane reporter CD4::mIFP together with markers for vesicles of the endocytic pathway. (E) Rab5::YFP, red arrowheads point to a Rab5‐positive vesicle; the white arrowhead shows the same vesicle once it lost the Rab5 signal. (F) FYVE::GFP, a PI_3_P reporter; and Spin::mRFP, a lysosomal marker. Black arrowheads point to small FYVE::GFP vesicles, and the squared area shows large ones at the tip. (G) Par6::mCherry and endogenously labelled Rab7::YFP. (H) Percentage of CD4::mIFP vesicles that carry the indicated markers. Data are plotted as mean ± SD. For each marker, we analysed at least 20 min of cell growth with 10–20 time points. Number of cells analysed: Par3, *n* = 2; Rab5, *n* = 3; FYVE::GFP, *n* = 4; FYVE::GFP‐Spin::RFP (which includes single‐ and double‐positive vesicles), *n* = 3; dLamp1, *n* = 2; Rab7, *n* = 5.Data information: Scale bars: 5 μm.Source data are available online for this figure.

Our observations point to a role for apical‐to‐basal transcytosis in coupling the extension of apical and basal membrane domains. The ideal way of investigating this experimentally would be to follow the path of plasma membrane lipids by live imaging. Unfortunately, no suitable probes exist that allow this in the developing tracheal system *in vivo*. Protein markers as proxies for membranes are not suitable to follow the full path because they are subject to sorting along the vesicle transport path. Instead, we studied individual segments of the potential path by imaging the movement of markers for well‐defined vesicular membrane compartments at high temporal resolution. Using CD4::mIFP, we found a large number of vesicles moving rapidly along the main axis of the growing terminal cell (Fig 7A, Movie EV7), often arising in the proximity of the tube and later moving towards the growing tip of the cell. Vesicle tracking revealed a net distal displacement regardless of where the vesicles originated (Fig 7B and C). This was not a consequence of cell elongation taking place in the same direction since the vesicles moved faster than the rate of tube elongation (Fig 7D).

**Figure EV4:**
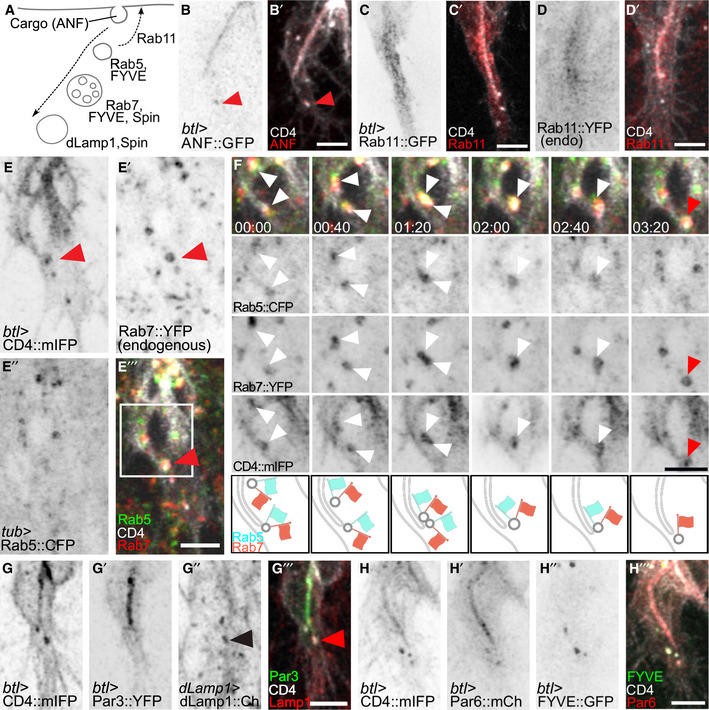
Distribution of late endosomal markers during terminal cell growth A–DTerminal cells expressing CD4::mIFP under *btl‐gal4,* together with markers of different compartments (summarized in A): (B) ANF::GFP; red arrowheads: a CD4::mIFP vesicle that carries ANF::GFP; (C) Rab11::GFP under *btl‐gal4*, (D) Endogenously YFP‐tagged Rab11.E, FCell expressing CD4::mIFP under *btl‐gal4,* Rab5::CFP under direct control of the tubulin promoter and endogenously tagged Rab7::YFP. (F) shows the area marked by the box in (E‴) at higher magnification and at six time points. White arrowheads: Rab5‐positive, Rab7‐positive CD4::mIFP vesicles; red arrowhead: Rab5‐negative, Rab7‐positive CD4::mIFP vesicle. A diagrammatic interpretation of the experiment shown in (E‐E‴) is shown below.G, HTerminal cells expressing CD4 together with (G) Par3::YFP and dLamp1::mCherry under its own promoter and (H) with Par6::mCherry and FYVE::GFP. Black and red arrowheads point to dLamp1::mCherry in a CD4 vesicle.Data information: Scale bars: 5 μm.

Vesicles first appearing in proximity to the tube suggested that they might be derived from the apical membrane, as was also indicated by the movement of the Par3‐positive vesicles (Appendix Fig S1). We simultaneously imaged CD4::mIFP with GFP fused to the rat atrial natriuretic factor signal peptide (ANF::GFP), an apically secreted cargo (Tsarouhas *et al*, 2007). Many CD4 vesicles were positive for ANF::GFP, suggesting that they emerged from the tube (Fig EV4A and B). In conclusion, we see extensive distal vesicle movement, and at least some of the vesicular membrane appears to be derived from the subcellular tube. To test whether these structures constitute part of the endocytic pathway, we analysed Rab5 distribution as a marker for early endosomes and found it was visible as discrete puncta around the tube, often localizing to CD4 vesicles. The association between Rab5 and CD4 vesicles was transient, with Rab5 being gradually lost from the vesicles over a few minutes (Fig 7E). This suggested that Rab5 endosomes were progressing into later endocytic compartments.

Early endosomes can be recycled back to the plasma membrane and this pathway is often employed to transcytose material from one compartment to another, in a process that relies on Rab11 (Pelissier *et al*, 2003; Bryant *et al*, 2010; Soulavie *et al*, 2018). If this pathway was used in the process we describe here, we would expect to see Rab11 associated with the Rab5‐positive structures near or at the CD4 vesicles. However, we found that both overexpressed Rab11 (this work and Gervais & Casanova, 2010) and endogenously tagged Rab11 formed clouds of small puncta that surrounded the tube and did not overlap with CD4 vesicles travelling to the tip (Fig EV4C and D).

Early endosomes can also undergo fusion and maturation into late endosomes. These steps involve replacement of Rab5 by Rab7 (Gillooly *et al*, 2001). Imaging Rab5, Rab7 and CD4 simultaneously showed several instances of CD4 vesicles containing both Rab5 and Rab7, and eventually losing Rab5 while retaining Rab7 (Fig EV4E and F). Rab5‐to‐7 conversion results in the recruitment of ESCRT components, leading to MVB formation and afterwards to the transition into lysosomes. We imaged a range of markers for different steps of the pathway: the FYVE domain of the ESCRT‐0 component Hrs (FYVE::GFP) for early endosomes and MVBs; the lysosomal permease Spinster (Spin::RFP), recruited in MVBs; and dLamp1 for lysosomes (Johnson *et al*, 2015; Riedel *et al*, 2016). Similar to Rab5, FYVE::GFP formed small puncta throughout the length of the tube (Fig 7F), but it was more prominently associated with the large CD4‐positive structures at the tip of the cell. Spin::RFP, dLamp::mCherry and Rab7 were seen almost exclusively in association with CD4 vesicles at the tip of the cell (Figs 7F and H, and EV4G). Given that the early markers Rab5 and FYVE::GFP were seen throughout the length of the cell, whereas the late markers Rab7, Spin and dLamp were restricted to the tip, we conclude that tube‐derived endosomes enter the late endosomal pathway during their movement towards the tip of the cell. This is consistent with our EM analyses where we found a polarized MVB distribution towards the tip of the cell.

To understand the relationship between the tube‐derived vesicles, the endocytic pathway and the apical membrane determinants at the tip of the cell, we studied the distribution of apical polarity proteins relative to late endocytic markers. FYVE::GFP, Rab7 and dLamp1 were often seen in proximity to apical proteins (Figs 7G and EV4G and H), suggesting that endocytic vesicles carrying cargo proteins are converted from early to late endosomes as they are displaced towards the tip of the cell.

### Dependence of large intracellular membrane structures on endocytosis

**Figure 8:**
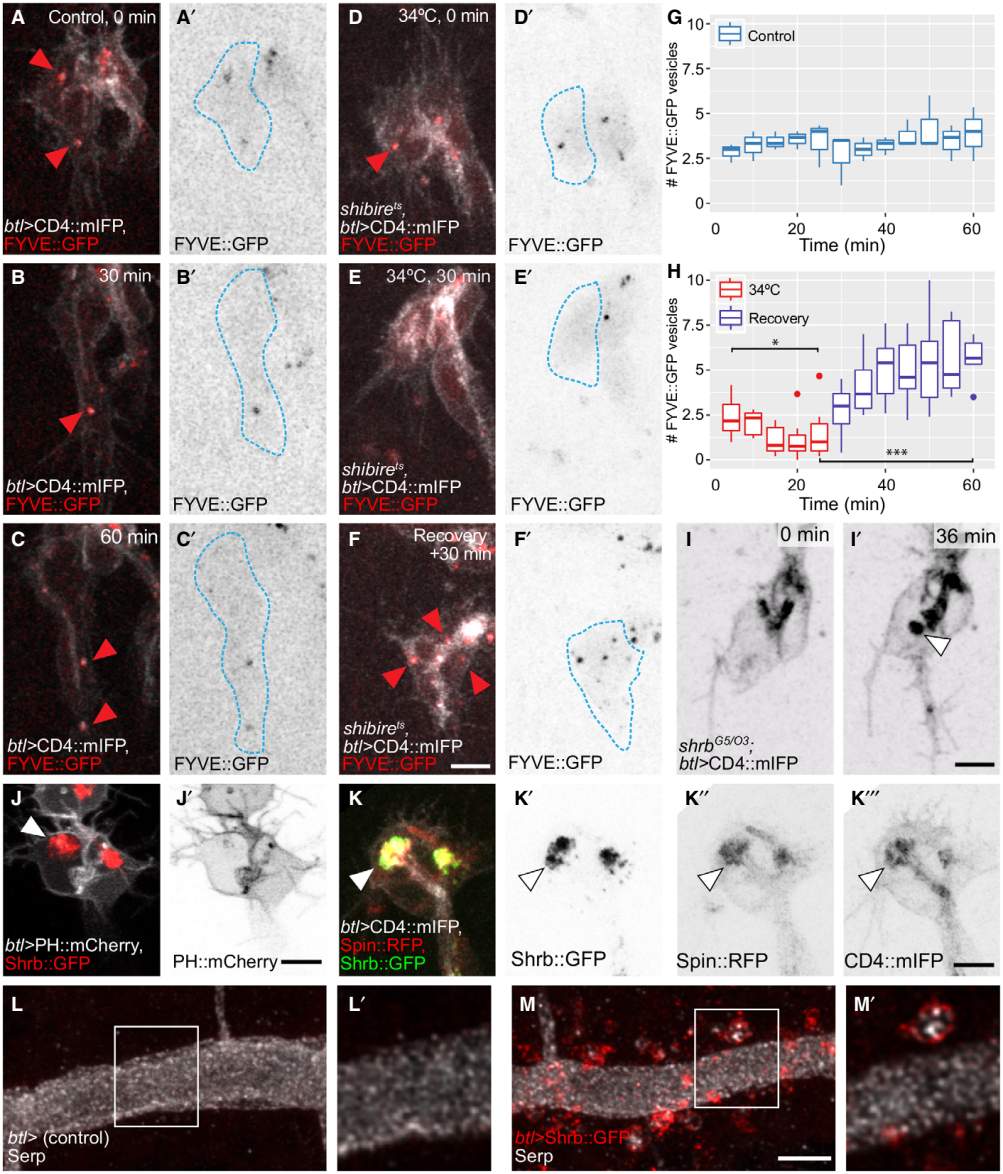
Distribution of late endosomal markers upon disruption of the endocytic pathway A–FTerminal cells expressing CD4::mIFP and FYVE::GFP under *btl‐gal4*. The outline of the cells was traced using the CD4::mIFP signal and is shown as a blue dashed line. (A–C) Control cell. (D–F) *shibire* ^*ts*^ cell before 34°C (D), after 30 min at 34°C (E) and after 30 min of recovery (F). Red arrowheads: FYVE::GFP puncta.G–HNumber of FYVE::GFP vesicles in 4 control cells (G) and in 6 *shibire* ^*ts*^ cells (H). Box plots represent median, interquartile range (IQR) and IQR*1.5 below and above the IQR. Significance in (H) was assessed using one‐way ANOVA with Geisser–Greenhouse correction for paired data and Dunnett's multiple comparison test. *P = 0.0288, ***P = 0.0016.I
*shrb* ^*O3/G5*^ mutant cell expressing CD4::mIFP under *btl‐gal4*. Arrowhead: CD4::mIFP aggregation.J, KTerminal cells overexpressing Shrb::GFP under *btl‐gal4* together with PH::mCherry (J) or together with Spin::RFP and CD4::mIFP (K). Arrowheads: Shrb::GFP accumulations.L, MDorsal trunk cells stained for Serp. In control embryos (L), Serp is seen exclusively in the tracheal lumen. The cells themselves are not visible in these images. In embryos expressing Shrb::GFP under *btl‐gal4* (M), Serp is also seen inside the cells, usually in association with or surrounded by Shrb::GFP. Boxed regions are magnified in (L′‐M′) and shown as single confocal planes.Data information: Scale bars: 5 μm.Source data are available online for this figure.

**Figure EV5:**
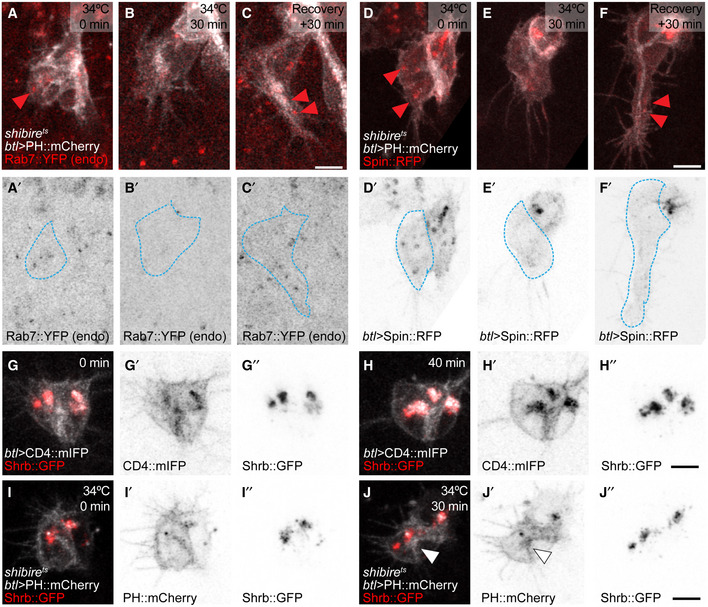
Effect on dynamin inactivation on vesicles carrying late endocytic markers A–F
*shibire* ^*ts*^ cells expressing PH::mCherry under *btl‐gal4*, together with endogenously labelled Rab7::YFP (A‐C) or with Spin::RFP (D‐F). The outlines of the terminal cells were traced using the mCherry fluorescence and superimposed on the image of the Rab7::YFP channel (blue broken line) to distinguish the cell from the surrounding tissue, which also expresses Rab7::YFP. Arrowheads point to Rab7 and Spin::RFP vesicles in the terminal cell.G, HTerminal cell expressing Shrb::GFP and CD4::mIFP under *btl‐gal4*, at the onset of tube formation (G) and 40 min later (H).I, J
*shibire* ^*ts*^ terminal cell expressing Shrb::GFP and PH::mCherry under *btl‐gal4* before dynamin inactivation (I) and after 30 min of inactivation (J). Arrowheads point to PH::mCherry accumulation in the apical compartment.Data information: Scale bars: 5 μm.

The results so far suggest that the late endosomes at the tip of the cell are sustained by material endocytosed at least in part from the tube membrane. If that was the case, blocking endocytosis should affect them. Quantification of FYVE::GFP‐positive vesicles showed that while control cells contained around 1–4 large FYVE::GFP‐CD4 vesicles at any given time, cells where dynamin was inactivated had very few, often none, of these vesicles (Fig 8A–E). Upon recovery, the number of FYVE::GFP‐CD4 vesicles was re‐established (Fig 8F–H) and even increased beyond the control conditions. We obtained similar results with Rab7::YFP and Spin::RFP (Fig EV5A–F). This indicates that the large membrane accumulations at the tip of the growing tracheal cell are formed from material delivered by endocytosis. This was consistent with our experiments on Crb and FGFR distribution: both were seen in vesicles in control cells, and these vesicles disappeared upon dynamin inactivation but reappeared during recovery (Fig 6B).

### Dependence of cell growth on MVB

If the destination of the majority of material endocytosed from the tube membrane is the compartment of late endosomal vesicles at the tip of the cell, the question arises where the membrane material moves from these structures, and whether any of it is delivered to the basal side. MVBs have been shown as a route for delivery of membrane and cargo to the plasma membrane (Zhang & Schekman, 2013; Dong *et al*, 2014a). Since in our experiments MVBs seem to act as collection points for apical and basal cargo, they may act as a hub for membrane redistribution to the basal membrane domain. To test this, we interfered with the proper biogenesis of the MVBs. MVB formation requires the function of Shrub (Shrb), the *Drosophila* homolog of ESCRT‐III component Snf7, and Shrb::GFP overexpression phenocopies *shrb* loss of function (Michelet *et al*, 2010; Dong *et al*, 2014a).

Terminal cells from *shrb* mutant embryos had a morphology similar to that caused by dynamin inactivation: the basal membrane failed to grow properly, and large accumulations of the CD4 membrane reporter were seen within the cells (Fig 8I). Shrb::GFP overexpression in wild‐type cells resulted in a similar, stronger phenotype (Fig EV5G and H). However, unlike in *shibire* ^*ts*^ cells, the aggregates that appear upon *shrb* loss of function did not contain PH::mCherry, indicating that they were not composed of plasma membrane (Fig 8J). Instead, the CD4::mIFP‐marked membrane accumulation also carried the late endosomal marker Spin::RFP (Fig 8K). Taken together, this shows that the membrane accumulated inside the cell does not correspond to an overgrown apical compartment, but rather represents membrane material that was *en route* to MVBs, but, because of impaired Shrb function, was not incorporated into MVBs or processed further.

The finding that in this case, neither the basal plasma membrane, nor the apical membrane showed any growth indicates that both depend on membrane trafficking through MVBs. For the basal membrane, this fits with the observation that its growth depends on endocytosis, but for apical membrane, this was unexpected. It can mean either of two things. All newly synthesized membrane could be directed through the MVB to the apical domain, and the apical domain therefore does not grow if MVBs are disrupted because it never receives membrane. Alternatively, membrane could be passed to the apical domain from the Golgi or the ER directly, but is completely recycled through endocytosis and passage through the MVB. We suggest that only the second scenario is consistent with our observations in *shibire* ^*ts*^ cells: the MVBs, as well as other vesicles carrying late endosomal markers, disappear in these cells, and yet the apical membrane continues to grow. This shows that the initial delivery of membrane to the apical domain does not require the MVBs. We also confirmed this by blocking endocytosis in cells overexpressing Shrb::GFP, where we found that material still accumulated in the apical domain even though MVBs were no longer functional (Fig EV5I and J).

These results show that in terminal cells, apical‐to‐basal transcytosis goes through MVBs, and we wondered if this was also true for other transcytotic routes. Serpentine (Serp) is a chitin deacetylase located in the tracheal lumen. It is produced in and secreted by tracheal cells early during embryogenesis, but at later stages, it is produced in the fat body and transported to the tracheal lumen in a process involving basal‐to‐apical transcytosis (Dong *et al*, 2014b). Serpentine is also endocytosed from and recycled back to the tracheal lumen (Dong *et al*, 2013). To test whether these processes depend on MVBs, we expressed Shrb::GFP in the tracheal system and looked at Serp distribution in the tracheal dorsal trunk. We found that in control embryos, Serp was only seen in the lumen (Fig 8L). In embryos expressing Shrb::GFP, Serp was also visible in cytoplasmic vesicles that were surrounded by Shrb::GFP itself (Fig 8M). This is consistent with results showing that *shrb* mutants accumulate Serp in tracheal cells (Dong *et al*, 2013). It is likely that Serp accumulation around Shrb::GFP is the result of basal‐to‐apical transcytosis and of apical‐to‐apical recycling of Serp in the dorsal trunk cells. Thus late endosomes also here appear to serve as stations that collect material from and re‐deliver it towards various compartments of the cell.

## Discussion

Our results suggest a route taken by plasma membrane material during the coordinated growth of the apical and basal domains of tracheal terminal cells, summarized in the synopsis image. We will discuss the evidence that leads us to postulate this path, and how it relates to previously discovered functions for components of vesicle trafficking systems and MVBs in cell morphogenesis and transcytosis.

### Initial delivery of membrane

We suggest that apical plasma membrane comes from the ER and Golgi, as also shown in MDCK cells in 3D cultures (Ferrari *et al*, 2008). In tracheal terminal cells, membrane is delivered throughout the length of the tube rather than any specific region (Gervais & Casanova, 2010). This fits with ER and Golgi being distributed along the entire length of the branch. We show that blocking MVB biogenesis prevents membrane delivery to either membrane compartment, which could argue for a role for MVBs in initial membrane delivery. However, we also show that MVB function is downstream of endocytosis.

### Retrieval by endocytosis

Endocytosis plays important roles at multiple steps of tracheal development (Tsarouhas *et al*, 2007; Schottenfeld‐Roames *et al*, 2014; Skouloudaki *et al*, 2019), but the fate of the membrane material ingested by the cell through apical endocytosis, the route it takes, and whether it contributes to elaborating the shape of the terminal cell were not known. In larval terminal cells, loss or reduction of endocytic components such as dynamin, Rab5 or syntaxin‐7 results in cysts in the apical membrane and sometimes in extreme curling of the apical membrane within branches. These cells also form fewer branches per cell (Schottenfeld‐Roames *et al*, 2014); thus, the phenotype suggests an increase in apical membrane at the expense of basal membrane. The presence of excess apical membrane correlated with raised levels of Crb and depended on Crb. In our experiments, even though Crb aggregated at the tube membrane, knocking it down did not prevent apical membrane accumulation. Furthermore, upon recovery of dynamin function, Crb levels were similar to controls. We conclude that here, Crb accumulation does not account for the apical increase resulting from endocytosis being blocked. The difference between the results in the embryo and the larva might be due to extremely different timescales: while we acutely blocked endocytosis for periods of 15 min to 2 h, larval cells that have grown for several days with reduced levels of endocytic components may be affected by compensatory processes, homeostatic regulation and longer‐term interaction between membrane delivery and Crb accumulation.

### Transcytosis

Transcytosis has been documented in many cell types, most commonly in the transport of proteins and other cargo through epithelial barriers, which can occur either from basal to apical, or vice versa (Sasaki *et al*, 2007; Gallet *et al*, 2008; Callejo *et al*, 2011; Yamazaki *et al*, 2016). The classical example is immunoglobulin secretion and uptake. In MDCK cells, transcytosis of immunoglobulins occurs also in both directions and relies on Rab17 and Rab25. However, similar gene duplications and diversifications have not occurred in *Drosophila* (Gramates *et al*, 2017; Fung *et al*, 2018).

Our conclusion that membrane material is transcytosed from the apical to the basal membrane domain rests on two observations: in the absence of endocytosis, the apical membrane continues to grow but the basal stops, and large‐scale physical connections appear between the basal and apical membrane after longer periods of blocking endocytosis. We view the sheets of membrane bridging the apical and basal membrane as evidence of membrane exchange between the two domains, representing structures that failed to be resolved normally in the absence of dynamin function. By this interpretation, unscissioned membrane invaginations protruding from the apical compartment, which are stripped of a clathrin coat, would occasionally have touched the basal plasma membrane or its protrusions and fused with it, as transcytosing vesicles would have done in the normal situation. Further plasma membrane delivery may then have expanded such initial channels into larger sheets. These membrane sheets also contain small vesicles similar to the ones produced by the ESCRT pathway to form MVBs, further supporting the delivery route we propose. Consistent with this model, severe dynamin inactivation in larval motor neurons results in a similar phenotype, with membrane cisternae bridging different regions of the synaptic bouton (Kasprowicz *et al*, 2014).

### Transcytosis as a morphogenetic mechanism

We are aware of only two cases where transcytosis is used as a morphogenetic mechanism: cellularization of the *Drosophila* blastoderm and morphogenesis of the excretory system in *C. elegans* (Pelissier *et al*, 2003; Soulavie *et al*, 2018). During cellularization, endocytosis retrieves membrane material from the highly folded apical plasma membrane which is subsequently delivered to the leading edge of the growing lateral membranes (Pelissier *et al*, 2003). The *C. elegans* duct cell of the excretory system forms a subcellular tube by wrapping and auto‐fusion of the plasma membrane. Basal‐to‐apical transcytosis is required for the elongation of this subcellular tube. This process does not depend on dynamin or clathrin but on AFF‐1, a protein that also mediates the auto‐fusion step (Sapir *et al*, 2007; Stone *et al*, 2009; Soulavie *et al*, 2018). Therefore, it is possible that the excretory duct cell adapted a different strategy to deliver membrane to build its subcellular tube, one that requires the AFF‐1 fusogen instead of clathrin/dynamin‐mediated endocytosis.

### MVBs in sorting and membrane delivery

We conclude that trafficking of membrane in the terminal cell goes through MVBs based on our functional results and the colocalization of a number of organelle, membrane and cargo markers. MVBs are known for their role in protein degradation, especially in the context of ligand‐bound receptors (Michelet *et al*, 2010). However, recycling membrane with its associated transmembrane proteins back to its site of origin forms part of that process, and some observations can be interpreted as transcytosing material being associated with MVBs. Gold‐labelled IgGs have been traced through various endosomal compartments, including MVBs, before they reach the basal membrane (He *et al*, 2008). Therefore, it is possible that newly synthesized proteins like FGFR reach MVBs but are then sorted out towards the basal compartment, while others like Crb are translocated into intraluminal vesicles and degraded, if not recycled back to the apical membrane. Observations in the dorsal trunk and in larval tracheal cells also indicate that this might be the case. Dorsal trunk tracheal cells lacking Shrb also accumulate Crb in late endosomes (Dong *et al*, 2014a). In larval terminal cells, loss of ESCRT‐0 leads to intracellular accumulation of FGFR and also to reduced FGF signalling (Chanut‐Delalande *et al*, 2010). According to our model, these results suggest that reduced FGF signalling activity in these cells is due to less FGFR reaching the basal plasma membrane. Thus, the defect is upstream of ligand–receptor interactions, rather than in signal transmission after receptor activation.

A multilayered membrane‐bound compartment seen in immature larval tracheal branches (Nikolova & Metzstein, 2015) has not been characterized in terms of marker distribution, but it is likely that it is functionally equivalent to the late endosomal compartments that we find at the growing tip of the embryonic terminal cell. Consistent with this, knockdown of vATPase components in larvae leads to formation of large intracellular Crb vesicles that are Rab5‐positive, enlarged Lamp1‐positive compartments and a distorted tube morphology (Francis & Ghabrial, 2015). Cells of the tracheal system that undergo anastomosis rely on specialized late endocytic vesicles called secretory lysosomes to drive tube fusion (Caviglia *et al*, 2016). These compartments are also positive for Rab7, and their secretion contributes to building a lumen between the fusing cells. MVBs may also be involved in the biogenesis of these vesicles; however, downstream mechanisms are likely to differ from the ones employed by terminal cells since fusion cells express a subset of proteins that have no role in terminal cell morphogenesis like Rab39 and the C2 domain protein Staccato (Caviglia *et al*, 2016).

In summary, our results show that plasma membrane turnover through the late endocytic pathway is a morphogenetic mechanism in which MVBs act as a hub for membrane and cargo sorting. This mechanism entails a massive plasma membrane flow that had so far not been documented. A role for MVBs in membrane homeostasis may be relevant for other cells with complex shapes, like neurons, which also rely heavily on polarized membrane trafficking to build long projections and to communicate with other tissues. While axons are generally devoid of MVBs, axon termini and dendrites frequently contain MVBs. MVBs have been proposed to serve as local stations to allow recycling, degradation or even storage of membrane material at a distance from the cell nucleus (Von Bartheld & Altick, 2011). Terminal cells, whose complexity increases dramatically within a few hours in terms of size and morphology, may represent an extreme case of membrane remodelling that relies on this mechanism, but this may nevertheless be required in the building of other complex cell shapes.

## Materials and Methods

### Fly lines

*UAS‐PLCδ‐PH‐Cherry* was generated by standard molecular biology techniques using UAS‐*PLCδ‐PH‐GFP* as template and cloned into pUAST‐attB. The construct was inserted in the VK00033 locus. *btl‐gal4* was used to drive UAS transgene expression in the trachea and was obtained from Markus Affolter laboratory, University of Basel, Switzerland (3rd chromosome), and Kyoto *Drosophila* Genetic Resource Center (#109128, 2nd chromosome). The following lines are from Bloomington: *UAS‐SrcGFP* (#5432), *UAS‐GalT‐GFP* (#30902), *UAS‐RFP‐KDEL* (#30909), *UAS‐IVS‐myr::tdTomato* (#32221), *UAS‐shrbGFP* (#32559)*, UAS‐GFP‐myc‐2xFYVE* and *UAS‐Spin::mRFP* (#42716), and *UAS‐CD4::mIFP‐T2A‐HO1* (#64182, #64183). From VDRC, we obtained *FGFR::GFP* (#318302) and *UAS‐crb‐IR* (#39177). We are grateful to the groups that kindly shared the following lines: endogenously labelled Rab7 and Rab11 (*YRab7* and *YRab11*), and Crb::GFP from Marko Brankatschk, TU Dresden, Germany (Dunst *et al*, 2015); *UAS‐CLC::GFP, UAS‐Shibire::YFP, UAS‐Rab5::YFP, UAS‐Rab11::GFP* and *shibire* ^*ts1*^ from Stefano De Renzis, EMBL, Germany (Fabrowski *et al*, 2013); *UAS‐3xeYFP‐Baz* (Par3::YFP) from Chris Doe, University of Oregon, USA (Siller *et al*, 2006); *tub‐Rab5::CFP* from Suzanne Eaton, MPI‐CBG, Germany (Marois *et al*, 2006); *dLamp::mCherry* from Gabor Juhasz, Eotvos Lorand University, Hungary (Hegedus *et al*, 2016); UAS‐*crb^extra^™::GFP* from Elisabeth Knust, MPI‐CBG, Germany (Johnson *et al*, 2002); *UAS‐PLCδ‐PH::GFP* from Thomas Lecuit, IBDM, France (JayaNandanan *et al*, 2014); *UAS‐palm::NeonGreen* from Stefan Luschnig, University of Münster, Germany (Sauerwald *et al*, 2017); *UAS‐ANF::GFP* and *UAS‐GASP::GFP* from Christos Samakovlis, Stockholm University, Sweden (Tsarouhas *et al*, 2007; Tiklova *et al*, 2013); and *UAS‐Par6::mCherry* from Daniel St Johnston, The Gurdon Institute, UK (Doerflinger *et al*, 2010). We used *shrb* null alleles from Bloomington, *shrb* ^*O3*^ (#39623) and *shrb* ^*G5*^ *(#39635)*. We balanced them over *CyO{dfd‐YFP}* and added *btl‐gal4, UAS‐CD4::mIFP* on the 3rd chromosome. *shrb* ^*O3*^ */shrb* ^*G5*^ embryos were selected by the absence of *dfd*‐YFP.

### Live imaging

Embryos were dechorionated in a 50% bleach solution, washed and mounted in halocarbon oil using glass coverslips or MatTek glass bottom dishes and heptane glue. Experiments at 34°C were done using a custom incubator chamber mounted on the microscope. For recovery experiments, embryos were mounted in water instead of oil, incubated at 34°C for the desired time, and for recovery, temperature was lowered to 23°C and ice‐cold water was added. Samples were imaged using a Zeiss LSM 880 in Airyscan Fast Mode, PerkinElmer UltraVIEW VoX or UltraVIEW ERS spinning disc confocal microscopes using Plan‐Apochromat 63×/1.4 Oil objectives. Airyscan images were deconvolved using the Zeiss ZEN software using the auto settings. Spinning disc images were deconvolved using Huygens Professional, SVI, and processed in FIJI. Unless otherwise stated, images are presented as maximum‐intensity z‐projections.

### Immunostainings

Embryos were dechorionated, devitellinized and fixed using 37% paraformaldehyde for 15 s while vortexing and 5 min in a rocker. Afterwards, embryos were blocked using bovine serum albumin and incubated overnight at 4°C in primary antibody solution, and secondary antibodies were incubated for 2 h the following day. Embryos were mounted in VECTASHIELD. We used the following antibodies: mouse anti‐βPS Integrin (1:200, DSHB #6G11), rat anti‐Crb (1:500, gift from Elisabeth Knust, MPI‐CBG, Germany), rat anti‐ECad (1:100, DSHB #DCAD2), mouse anti‐FasIII (1:200 DSHB #7G10), mouse anti‐dpERK (1:200, Sigma‐Aldrich #M8159), rabbit anti‐Dof [1:200 (Vincent *et al*, 1998)] and rabbit anti‐Serp (1:300, gift from Stefan Luschnig, University of Münster, Germany). CBD conjugated to Alexa 647 was described previously (JayaNandanan *et al*, 2014). To enhance signal from fluorescent reporters, we used GFP‐booster coupled to Atto488 (gba488) and RFP‐booster coupled to Atto594 (rba594) from Chromotek. Secondary antibodies used were from Thermo Scientific: Alexa 568 goat anti‐mouse (1:500, A‐11031), Alexa 568 goat anti‐rat (1:500, A‐11077) or Alexa 647 goat anti‐rabbit (1:300, A‐21245). Embryos were imaged using a Zeiss LSM 780 using a Plan‐Apochromat 63×/1.4 Oil objective and deconvolved with Huygens Professional and processed in FIJI. Unless otherwise stated, images are presented as maximum‐intensity z‐projections.

### Electron tomography

#### Initial EM workflow

The embryos were staged and high‐pressure frozen (HPM010 Abra Fluid) in 20% Ficoll (mol weight ~70,000). The freeze‐substitution was done (EM‐AFS2, Leica Microsystems) with 0.3% uranyl acetate, 0.3% glutaraldehyde and 3% water in acetone at −90°C for 48 h. The temperature was then raised to −45°C at 3.5°C/h, and samples were further incubated for 5 h. The samples were rinsed in acetone, followed by infiltration in Lowicryl HM20 resin, while raising the temperature to −25°C. The samples were polymerized under UV light for 48 h at −25°C and for further 9 h, while the temperature was gradually raised to 20°C (5°C/h). Thick sections (300 nm) were cut from the polymerized resin block and picked up on carbon and Formvar‐coated slot grids.

The sections were screened using a FEI Tecnai F30 electron microscope with a Gatan OneView camera and acquiring large field‐of‐view montages with SerialEM (summarized in Appendix Fig S2). The serial section montages were aligned and segmented using the IMOD package (Mastronarde, 1997) in order to find terminal cells. The sections that had terminal cells were then imaged again for electron tomography.

#### CLEM workflow

High‐pressure freezing and freeze‐substitution were done as above, except that freeze‐substitution was done with 0.1% uranyl acetate in acetone at −90°C for 48 h. The fluorescence microscopy imaging of the sections was carried out as previously described (Hampoelz *et al*, 2016) using a widefield fluorescence microscope (Olympus IX81). The images collected were used to screen for the sections with terminal cells. Those sections were then used for electron tomography. For electron tomography, tilt series were performed in 1 degree increments from 60 to −60 degrees with 2.549 nm or 0.78 nm pixel size on a FEI Tecnai F30 electron microscope with a Gatan OneView camera. The serial tomograms were reconstructed, aligned and segmented using the IMOD package.

### Image analyses

Tube length and vesicle displacement were analysed in FIJI using the manual tracking plug‐in. Organelle distance from the base of the cell was measured manually in FIJI, setting the junction to the previous cell as origin and measuring the distance along the tube to the tip of the cell.

To determine the total amount of membrane present in the cells, we did z SUM projections and then determined background signal outside the cell and subtracted this from the pixel values in the SUM. We manually segmented the cell and the tube within the cell. To determine the fluorescence intensity of the apical membrane, we measured the total pixel intensity values (RawIntDen) within the apical membrane. The z SUM in this area also includes signal from the basal membrane below and above the apical tube. We determined this amount by measuring an area next to the tube and subtracted this value from the one we had measured for the tube. We took the resulting value to be the membrane in the apical domain. To determine the amount of membrane in the basal domain, we therefore simply subtracted the apical value from the total value.

To quantify the amount of βPS‐integrin at the apical membrane compartment, we subtracted background, manually segmented the tube within the cell from z SUM substacks using FIJI and quantified the pixel values for βPS‐integrin within it. The values were normalized to the mean pixel intensity values surrounding the cell. For Crb, we used a similar approach but instead we measured the pixel values at the apical membrane of the dorsal trunk cells and normalized them to the signal to the values of the epidermis. Marker occupancy at CD4 vesicles and number of vesicles in time were scored manually in FIJI.

FGFR and dpERK signal quantifications were done by volume in Imaris, using the Dof signal as 3D mask. Since Dof is a cytoplasmic protein and it does not label filopodia, using it for segmentation allowed us to discard the amount of FGFR still present in the basal plasma membrane.

EM images were analysed using the IMOD package. The number of vesicles on a given tomogram was normalized to the area covered by cytoplasm at the mid z‐plane of the tomogram. Large vesicles like MVBs can span more than one tomogram, and we ensured these were only counted once whenever they were visible in more than one tomogram. Endocytic events were normalized to the surface of plasma membrane visible on a given tomogram.

### Statistical analyses

We used GraphPad Prism 6 for all statistical analyses. Plots were generated using RStudio and the ggplot2 package.

## Author contributions

MR, R‐BLD and LM conceived the study. MR, R‐BLD and MP contributed to methodology and investigation. R‐BLD performed formal analysis and wrote the original draft of the manuscript. MR, R‐BLD, MP, SY and LM wrote, reviewed and edited the manuscript. R‐BLD contributed to visualization. SY and LM supervised the study. LM acquired funding.

## Conflict of interest

Maria Leptin is director of EMBO. *The EMBO Journal* is editorially independent of EMBO.

## Supporting information



Appendix

Expanded View Figures PDF

Movie EV1

Movie EV2

Movie EV3

Movie EV4

Movie EV5

Movie EV6

Movie EV7

Source Data for Expanded View

Review Process File

Source Data for Figure 1

Source Data for Figure 3

Source Data for Figure 4

Source Data for Figure 6

Source Data for Figure 7

Source Data for Figure 8

## Data Availability

The electron microscopy tomograms presented in Figs 2, 3 and 5 have been deposited at the Electron Microscopy Public Image Archive (EMPIAR, https://www.ebi.ac.uk/pdbe/emdb/empiar/) with the accession number EMPIAR‐10434.

## References

[embj2020105332-bib-0001] Baer MM , Palm W , Eaton S , Leptin M , Affolter M (2012) Microsomal triacylglycerol transfer protein (MTP) is required to expand tracheal lumen in *Drosophila* in a cell‐autonomous manner. J Cell Sci 125: 6038–6048 23132924 10.1242/jcs.110452

[embj2020105332-bib-0002] Balleza E , Kim JM , Cluzel P (2018) Systematic characterization of maturation time of fluorescent proteins in living cells. Nat Methods 15: 47–51 29320486 10.1038/nmeth.4509 PMC5765880

[embj2020105332-bib-0003] Bellec K , Gicquel I , Le Borgne R (2018) Stratum recruits Rab8 at Golgi exit sites to regulate the basolateral sorting of Notch and Sanpodo. Development 145: dev163469 29967125 10.1242/dev.163469

[embj2020105332-bib-0004] Bryant DM , Datta A , Rodriguez‐Fraticelli AE , Peranen J , Martin‐Belmonte F , Mostov KE (2010) A molecular network for *de novo* generation of the apical surface and lumen. Nat Cell Biol 12: 1035–1045 20890297 10.1038/ncb2106 PMC2975675

[embj2020105332-bib-0005] Callejo A , Bilioni A , Mollica E , Gorfinkiel N , Andres G , Ibanez C , Torroja C , Doglio L , Sierra J , Guerrero I (2011) Dispatched mediates Hedgehog basolateral release to form the long‐range morphogenetic gradient in the *Drosophila* wing disk epithelium. Proc Natl Acad Sci USA 108: 12591–12598 21690386 10.1073/pnas.1106881108 PMC3150953

[embj2020105332-bib-0006] Caviglia S , Brankatschk M , Fischer EJ , Eaton S , Luschnig S (2016) Staccato/Unc‐13‐4 controls secretory lysosome‐mediated lumen fusion during epithelial tube anastomosis. Nat Cell Biol 18: 727–739 27323327 10.1038/ncb3374

[embj2020105332-bib-0007] Chanut‐Delalande H , Jung AC , Baer MM , Lin L , Payre F , Affolter M (2010) The Hrs/Stam complex acts as a positive and negative regulator of RTK signaling during *Drosophila* development. PLoS ONE 5: e10245 20422006 10.1371/journal.pone.0010245 PMC2858154

[embj2020105332-bib-0008] Deborde S , Perret E , Gravotta D , Deora A , Salvarezza S , Schreiner R , Rodriguez‐Boulan E (2008) Clathrin is a key regulator of basolateral polarity. Nature 452: 719–U3 18401403 10.1038/nature06828 PMC4078870

[embj2020105332-bib-0009] Doerflinger H , Vogt N , Torres IL , Mirouse V , Koch I , Nusslein‐Volhard C , St Johnston D (2010) Bazooka is required for polarisation of the *Drosophila* anterior‐posterior axis. Development 137: 1765–1773 20430751 10.1242/dev.045807 PMC2860254

[embj2020105332-bib-0010] Dong B , Kakihara K , Otani T , Wada H , Hayashi S (2013) Rab9 and retromer regulate retrograde trafficking of luminal protein required for epithelial tube length control. Nat Commun 4: 1312–1358 10.1038/ncomms2347 PMC3562448 23322046

[embj2020105332-bib-0011] Dong B , Hannezo E , Hayashi S (2014a) Balance between apical membrane growth and luminal matrix resistance determines epithelial tubule shape. Cell Rep 7: 941–950 24794438 10.1016/j.celrep.2014.03.066

[embj2020105332-bib-0012] Dong B , Miao G , Hayashi S (2014b) A fat body‐derived apical extracellular matrix enzyme is transported to the tracheal lumen and is required for tube morphogenesis in *Drosophila* . Development 141: 4104–4109 25336738 10.1242/dev.109975 PMC4302886

[embj2020105332-bib-0013] Dunst S , Kazimiers T , von Zadow F , Jambor H , Sagner A , Brankatschk B , Mahmoud A , Spannl S , Tomancak P , Eaton S *et al* (2015) Endogenously tagged rab proteins: a resource to study membrane trafficking in *Drosophila* . Dev Cell 33: 351–365 25942626 10.1016/j.devcel.2015.03.022 PMC4431667

[embj2020105332-bib-0502] Fabrowski P , Necakov AS , Mumbauer S , Loeser E , Reversi A , Streichan S , Briggs JA , De Renzis S (2013) Tubular endocytosis drives remodelling of the apical surface during epithelial morphogenesis in Drosophila. Nat Commun 4: 2244 23921440 10.1038/ncomms3244 PMC3753550

[embj2020105332-bib-0014] Ferrari A , Veligodskiy A , Berge U , Lucas MS , Kroschewski R (2008) ROCK‐mediated contractility, tight junctions and channels contribute to the conversion of a preapical patch into apical surface during isochoric lumen initiation. J Cell Sci 121: 3649–3663 18946028 10.1242/jcs.018648

[embj2020105332-bib-0015] Francis D , Ghabrial AS (2015) Compensatory branching morphogenesis of stalk cells in the *Drosophila* trachea. Development 142: 2048–2057 25977367 10.1242/dev.119602 PMC4460740

[embj2020105332-bib-0016] Fung KYY , Fairn GD , Lee WL (2018) Transcellular vesicular transport in epithelial and endothelial cells: challenges and opportunities. Traffic 19: 5–18 28985008 10.1111/tra.12533

[embj2020105332-bib-0017] Gabay L , Seger R , Shilo BZ (1997) MAP kinase *in situ* activation atlas during *Drosophila* embryogenesis. Development 124: 3535–3541 9342046 10.1242/dev.124.18.3535

[embj2020105332-bib-0018] Gallet A , Staccini‐Lavenant L , Therond PP (2008) Cellular trafficking of the glypican Dally‐like is required for full‐strength Hedgehog signaling and wingless transcytosis. Dev Cell 14: 712–725 18477454 10.1016/j.devcel.2008.03.001

[embj2020105332-bib-0019] Gervais L , Casanova J (2010) *In vivo* coupling of cell elongation and lumen formation in a single cell. Curr Biol 20: 359–366 20137948 10.1016/j.cub.2009.12.043

[embj2020105332-bib-0020] Ghabrial AS , Levi BP , Krasnow MA (2011) A systematic screen for tube morphogenesis and branching genes in the *Drosophila* tracheal system. PLoS Genet 7: e1002087 21750678 10.1371/journal.pgen.1002087 PMC3131284

[embj2020105332-bib-0021] Gillooly DJ , Simonsen A , Stenmark H (2001) Cellular functions of phosphatidylinositol 3‐phosphate and FYVE domain proteins. Biochem J 355: 249–258 11284710 10.1042/0264-6021:3550249 PMC1221734

[embj2020105332-bib-0022] Gramates LS , Marygold SJ , Santos GD , Urbano JM , Antonazzo G , Matthews BB , Rey AJ , Tabone CJ , Crosby MA , Emmert DB *et al* (2017) FlyBase at 25: looking to the future. Nucleic Acids Res 45: D663–D671 27799470 10.1093/nar/gkw1016 PMC5210523

[embj2020105332-bib-0023] Guo Y , Sirkis DW , Schekman R (2014) Protein sorting at the trans ‐Golgi network. Annu Rev Cell Dev Biol 30: 169–206 25150009 10.1146/annurev-cellbio-100913-013012

[embj2020105332-bib-0501] Hampoelz B , Mackmull MT , Machado P , Ronchi P , Bui KH , Schieber N , Santarella‐Mellwig R , Necakov A , Andres‐Pons A , Philippe JM *et al* (2016) Pre‐assembled Nuclear Pores Insert into the Nuclear Envelope during Early Development. Cell 166: 664–678 27397507 10.1016/j.cell.2016.06.015 PMC4967450

[embj2020105332-bib-0024] He W , Ladinsky MS , Huey‐Tubman KE , Jensen GJ , McIntosh JR , Bjorkman PJ (2008) FcRn‐mediated antibody transport across epithelial cells revealed by electron tomography. Nature 455: 542–546 18818657 10.1038/nature07255 PMC2773227

[embj2020105332-bib-0025] Hegedus K , Takats S , Boda A , Jipa A , Nagy P , Varga K , Kovacs AL , Juhasz G (2016) The Ccz1‐Mon1‐Rab7 module and Rab5 control distinct steps of autophagy. Mol Biol Cell 27: 3132–3142 27559127 10.1091/mbc.E16-03-0205 PMC5063620

[embj2020105332-bib-0026] Holthuis JC , Menon AK (2014) Lipid landscapes and pipelines in membrane homeostasis. Nature 510: 48–57 24899304 10.1038/nature13474

[embj2020105332-bib-0027] JayaNandanan N , Mathew R , Leptin M (2014) Guidance of subcellular tubulogenesis by actin under the control of a synaptotagmin‐like protein and Moesin. Nat Commun 5: 3036 24413568 10.1038/ncomms4036 PMC3945880

[embj2020105332-bib-0028] Johnson K , Grawe F , Grzeschik N , Knust E (2002) *Drosophila* crumbs is required to inhibit light‐induced photoreceptor degeneration. Curr Biol 12: 1675–1680 12361571 10.1016/s0960-9822(02)01180-6

[embj2020105332-bib-0029] Johnson AE , Shu H , Hauswirth AG , Tong A , Davis GW (2015) VCP‐dependent muscle degeneration is linked to defects in a dynamic tubular lysosomal network *in vivo* . Elife 4: e07366 10.7554/eLife.07366 PMC4574298 26167652

[embj2020105332-bib-0030] Jones TA , Nikolova LS , Schjelderup A , Metzstein MM (2014) Exocyst‐mediated membrane trafficking is required for branch outgrowth in *Drosophila* tracheal terminal cells. Dev Biol 390: 41–50 24607370 10.1016/j.ydbio.2014.02.021 PMC4041209

[embj2020105332-bib-0031] Kasprowicz J , Kuenen S , Swerts J , Miskiewicz K , Verstreken P (2014) Dynamin photoinactivation blocks Clathrin and alpha‐adaptin recruitment and induces bulk membrane retrieval. J Cell Biol 204: 1141–1156 24662566 10.1083/jcb.201310090 PMC3971740

[embj2020105332-bib-0032] Koenig JH , Ikeda K (1989) Disappearance and reformation of synaptic vesicle membrane upon transmitter release observed under reversible blockage of membrane retrieval. J Neurosci 9: 3844–3860 2573698 10.1523/JNEUROSCI.09-11-03844.1989 PMC6569944

[embj2020105332-bib-0033] Kukulski W , Schorb M , Welsch S , Picco A , Kaksonen M , Briggs JAG (2011) Correlated fluorescence and 3D electron microscopy with high sensitivity and spatial precision. J Cell Biol 192: 111–119 21200030 10.1083/jcb.201009037 PMC3019550

[embj2020105332-bib-0034] Lerner DW , McCoy D , Isabella AJ , Mahowald AP , Gerlach GF , Chaudhry TA , Horne‐Badovinac S (2013) A Rab10‐dependent mechanism for polarized basement membrane secretion during organ morphogenesis. Dev Cell 24: 159–168 23369713 10.1016/j.devcel.2012.12.005 PMC3562474

[embj2020105332-bib-0035] Marois E , Mahmoud A , Eaton S (2006) The endocytic pathway and formation of the Wingless morphogen gradient. Development 133: 307–317 16354714 10.1242/dev.02197

[embj2020105332-bib-0036] Mastronarde DN (1997) Dual‐axis tomography: an approach with alignment methods that preserve resolution. J Struct Biol 120: 343–352 9441937 10.1006/jsbi.1997.3919

[embj2020105332-bib-0037] Michelet X , Djeddi A , Legouis R (2010) Developmental and cellular functions of the ESCRT machinery in pluricellular organisms. Biol Cell 102: 191–202 20059450 10.1042/BC20090145

[embj2020105332-bib-0038] Nikolova LS , Metzstein MM (2015) Intracellular lumen formation in *Drosophila* proceeds via a novel subcellular compartment. Development 142: 3964–3973 26428009 10.1242/dev.127902 PMC6517834

[embj2020105332-bib-0039] Nixon SJ , Webb RI , Floetenmeyer M , Schieber N , Lo HP , Parton RG (2009) A single method for cryofixation and correlative light, electron microscopy and tomography of zebrafish embryos. Traffic 10.1111/j.1600-0854.2008.00859.x 19054388

[embj2020105332-bib-0040] Okenve‐Ramos P , Llimargas M (2014) Fascin links Btl/FGFR signalling to the actin cytoskeleton during *Drosophila* tracheal morphogenesis. Development 141: 929–939 24496629 10.1242/dev.103218

[embj2020105332-bib-0041] Pelissier A , Chauvin J‐P , Lecuit T (2003) Trafficking through Rab11 endosomes is required for cellularization during *Drosophila* embryogenesis. Curr Biol 13: 1848–1857 14588240 10.1016/j.cub.2003.10.023

[embj2020105332-bib-0042] Pellikka M , Tanentzapf G , Pinto M , Smith C , McGlade CJ , Ready DF , Tepass U (2002) Crumbs, the *Drosophila* homologue of human CRB1/RP12, is essential for photoreceptor morphogenesis. Nature 416: 143–149 11850625 10.1038/nature721

[embj2020105332-bib-0043] Ricolo D , Deligiannaki M , Casanova J , Araujo SJ (2016) Centrosome amplification increases single‐cell branching in post‐mitotic cells. Curr Biol 26: 2805–2813 27693136 10.1016/j.cub.2016.08.020

[embj2020105332-bib-0044] Riedel F , Gillingham AK , Rosa‐Ferreira C , Galindo A , Munro S (2016) An antibody toolkit for the study of membrane traffic in *Drosophila* melanogaster. Biol Open 5: 987–992 27256406 10.1242/bio.018937 PMC4958275

[embj2020105332-bib-0045] Rios‐Barrera LD , Sigurbjörnsdóttir S , Baer M , Leptin M (2017) Dual function for Tango1 in secretion of bulky cargo and in ER‐Golgi morphology. Proc Natl Acad Sci U S A 114: E10389–E10398 29138315 10.1073/pnas.1711408114 PMC5715762

[embj2020105332-bib-0046] Saheki Y , De Camilli P (2017) Endoplasmic reticulum‐plasma membrane contact sites. Annu Rev Biochem 86: 659–684 28301744 10.1146/annurev-biochem-061516-044932

[embj2020105332-bib-0047] Sapir A , Choi J , Leikina E , Avinoam O , Valansi C , Chernomordik LV , Newman AP , Podbilewicz B (2007) AFF‐1, a FOS‐1‐regulated fusogen, mediates fusion of the anchor cell in *C. elegans* . Dev Cell 12: 683–698 17488621 10.1016/j.devcel.2007.03.003 PMC1975806

[embj2020105332-bib-0048] Sasaki N , Sasamura T , Ishikawa HO , Kanai M , Ueda R , Saigo K , Matsuno K (2007) Polarized exocytosis and transcytosis of Notch during its apical localization in *Drosophila* epithelial cells. Genes Cells 12: 89–103 17212657 10.1111/j.1365-2443.2007.01037.x

[embj2020105332-bib-0049] Sauerwald J , Soneson C , Robinson MD , Luschnig S (2017) Faithful mRNA splicing depends on the Prp19 complex subunit faint sausage and is required for tracheal branching morphogenesis in *Drosophila* . Development 144: 657–663 28087625 10.1242/dev.144535

[embj2020105332-bib-0050] Schottenfeld‐Roames J , Ghabrial AS (2013) Osmotic regulation of seamless tube growth. Nat Cell Biol 15: 137–139 23377027 10.1038/ncb2683 PMC4520389

[embj2020105332-bib-0051] Schottenfeld‐Roames J , Rosa JB , Ghabrial AS (2014) Seamless tube shape is constrained by endocytosis‐dependent regulation of active Moesin. Curr Biol 24: 1756–1764 25065756 10.1016/j.cub.2014.06.029 PMC4125418

[embj2020105332-bib-0052] Sigurbjörnsdóttir S , Mathew R , Leptin M (2014) Molecular mechanisms of *de novo* lumen formation. Nat Rev Mol Cell Biol 15: 665–676 25186133 10.1038/nrm3871

[embj2020105332-bib-0053] Siller KH , Cabernard C , Doe CQ (2006) The NuMA‐related Mud protein binds Pins and regulates spindle orientation in *Drosophila* neuroblasts. Nat Cell Biol 8: 594–600 16648843 10.1038/ncb1412

[embj2020105332-bib-0054] Skouloudaki K , Papadopoulos DK , Tomancak P , Knust E (2019) The apical protein Apnoia interacts with Crumbs to regulate tracheal growth and inflation. PLoS Genet 15: e1007852 30645584 10.1371/journal.pgen.1007852 PMC6333334

[embj2020105332-bib-0055] Soulavie F , Hall DH , Sundaram MV (2018) The AFF‐1 exoplasmic fusogen is required for endocytic scission and seamless tube elongation. Nat Commun 9: 1741 29717108 10.1038/s41467-018-04091-1 PMC5931541

[embj2020105332-bib-0056] Stone CE , Hall DH , Sundaram MV (2009) Lipocalin signaling controls unicellular tube development in the *Caenorhabditis elegans* excretory system. Dev Biol 329: 201–211 19269285 10.1016/j.ydbio.2009.02.030 PMC3030807

[embj2020105332-bib-0057] Tiklova K , Tsarouhas V , Samakovlis C (2013) Control of airway tube diameter and integrity by secreted chitin‐binding proteins in *Drosophila* . PLoS ONE 8: e67415 23826295 10.1371/journal.pone.0067415 PMC3691276

[embj2020105332-bib-0058] Tsarouhas V , Senti KA , Jayaram SA , Tiklova K , Hemphala J , Adler J , Samakovlis C (2007) Sequential pulses of apical epithelial secretion and endocytosis drive airway maturation in *Drosophila* . Dev Cell 13: 214–225 17681133 10.1016/j.devcel.2007.06.008

[embj2020105332-bib-0059] Villaseñor R , Kalaidzidis Y , Zerial M (2016) Signal processing by the endosomal system. Curr Opin Cell Biol 39: 53–60 26921695 10.1016/j.ceb.2016.02.002

[embj2020105332-bib-0503] Vincent S , Wilson R , Coelho C , Affolter M , Leptin M (1998) The Drosophila protein Dof is specifically required for FGF signaling. Mol. Cell 2: 515–525 9809073 10.1016/s1097-2765(00)80151-3

[embj2020105332-bib-0060] Von Bartheld CS , Altick AL (2011) Multivesicular bodies in neurons: distribution, protein content, and trafficking functions. Prog Neurobiol 93: 313–340 21216273 10.1016/j.pneurobio.2011.01.003 PMC3055956

[embj2020105332-bib-0061] Wilson PD (2011) Apico‐basal polarity in polycystic kidney disease epithelia. Biochim Biophys Acta 1812: 1239–1248 21658447 10.1016/j.bbadis.2011.05.008

[embj2020105332-bib-0062] Wodarz A , Hinz U , Engelbert M , Knust E (1995) Expression of crumbs confers apical character on plasma membrane domains of ectodermal epithelia of *Drosophila* . Cell 82: 67–76 7606787 10.1016/0092-8674(95)90053-5

[embj2020105332-bib-0063] Wu B , Guo W (2015) The exocyst at a glance. J Cell Sci 128: 2957–2964 26240175 10.1242/jcs.156398 PMC4541039

[embj2020105332-bib-0064] Yamazaki Y , Palmer L , Alexandre C , Kakugawa S , Beckett K , Gaugue I , Palmer RH , Vincent JP (2016) Godzilla‐dependent transcytosis promotes Wingless signalling in *Drosophila* wing imaginal discs. Nat Cell Biol 18: 451–457 26974662 10.1038/ncb3325 PMC4817240

[embj2020105332-bib-0065] Yu D , Baird MA , Allen JR , Howe ES , Klassen MP , Reade A , Makhijani K , Song Y , Liu S , Murthy Z *et al* (2015) A naturally monomeric infrared fluorescent protein for protein labeling *in vivo* . Nat Methods 12: 763–765 26098020 10.1038/nmeth.3447 PMC4521985

[embj2020105332-bib-0066] Zang Y , Wan M , Liu M , Ke H , Ma S , Liu LP , Ni JQ , Pastor‐Pareja JC (2015) Plasma membrane overgrowth causes fibrotic collagen accumulation and immune activation in *Drosophila* adipocytes. Elife 4: e07187 26090908 10.7554/eLife.07187 PMC4490375

[embj2020105332-bib-0067] Zhang M , Schekman R (2013) Cell biology. Unconventional secretion, unconventional solutions. Science 340: 559–561 23641104 10.1126/science.1234740

