# Supplementary material for: Transcytosis via the late endocytic pathway as a cell morphogenetic mechanism
Source: EMBO J. 2020 Jul 13;39(16):e105332. doi: 10.15252/embj.2020105332 (PMC7429744; doi:10.15252/embj.2020105332)
Supplement: Supplementary file 1 — Appendix [file EMBJ-39-e105332-s001.docx]

Appendix

Table of contents

1. Appendix Figure S1. Distribution and composition of membrane reporters during tube morphogenesis
2. Appendix Figure S2. Effect of blocking endocytosis on membrane proteins.

Appendix Figure S1. Distribution and composition of membrane reporters during tube morphogenesis.


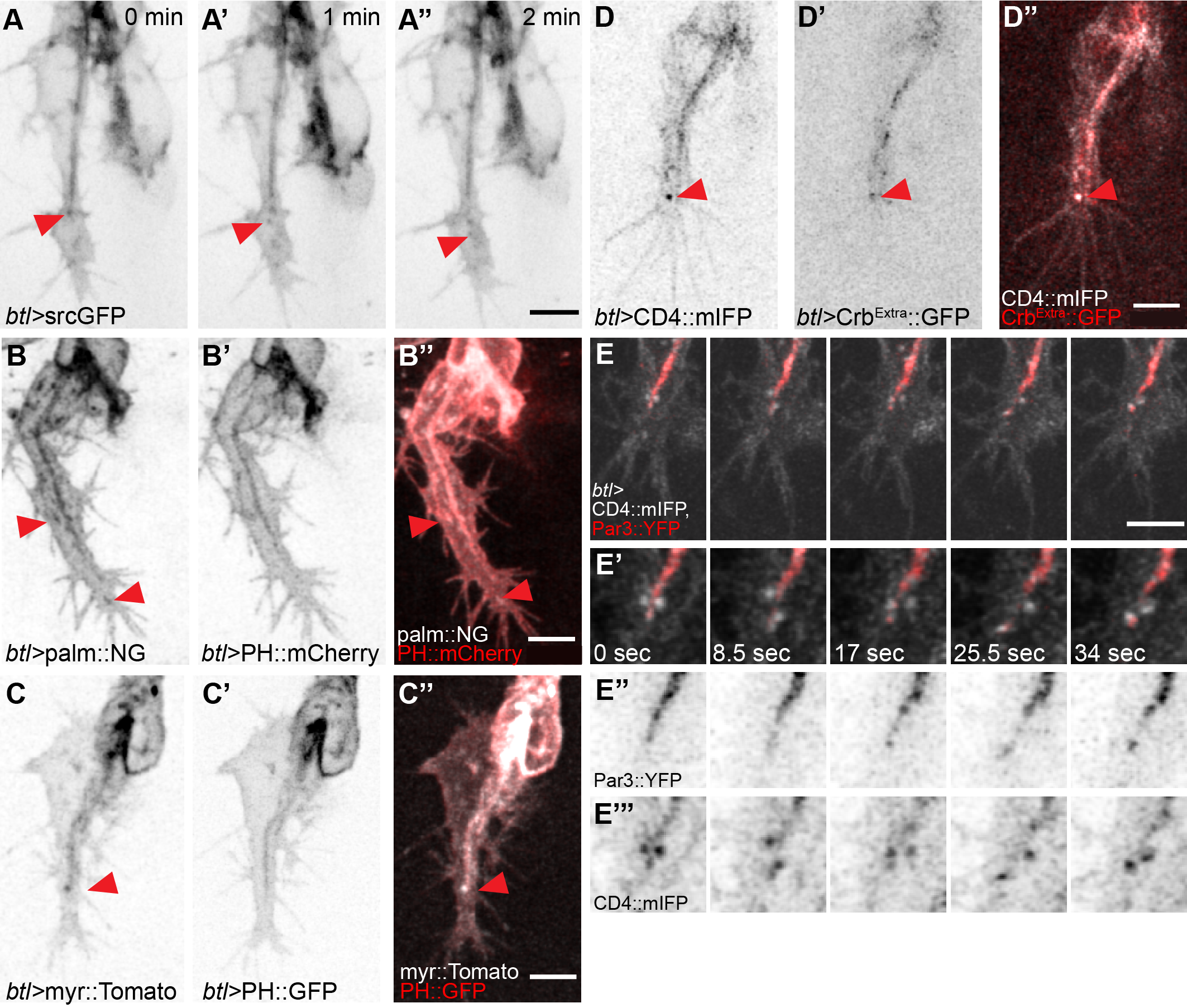


(A-C) Terminal cells expressing membrane reporters: (A-A’’) GFP fused to the myristoylation signal of Src, srcGFP; (B-B’’) Palmitoylated Neon Green (palm::NG, B’) and the PH domain of PLCδ fused to mCherry (PH::mCherry, B’); (C-C’’) myristoylated Tomato (myr::Tomato, C) and the PH domain of PLCδ fused to GFP (PH::GFP; C’). (D-E) Terminal cells expressing the general plasma membrane marker CD4::mIFP in combination with (D-D’’) the extracellular domain of Crb fused to GFP (Crb^Extra^::GFP, D’), and with Par3::YFP imaged at high temporal resolution (E-E’’’). Red arrowheads in (A-D) show vesicles and associated markers at the tip of the cell. Scale bars: 5μm

Appendix Figure S2. Effect of blocking endocytosis on membrane proteins.


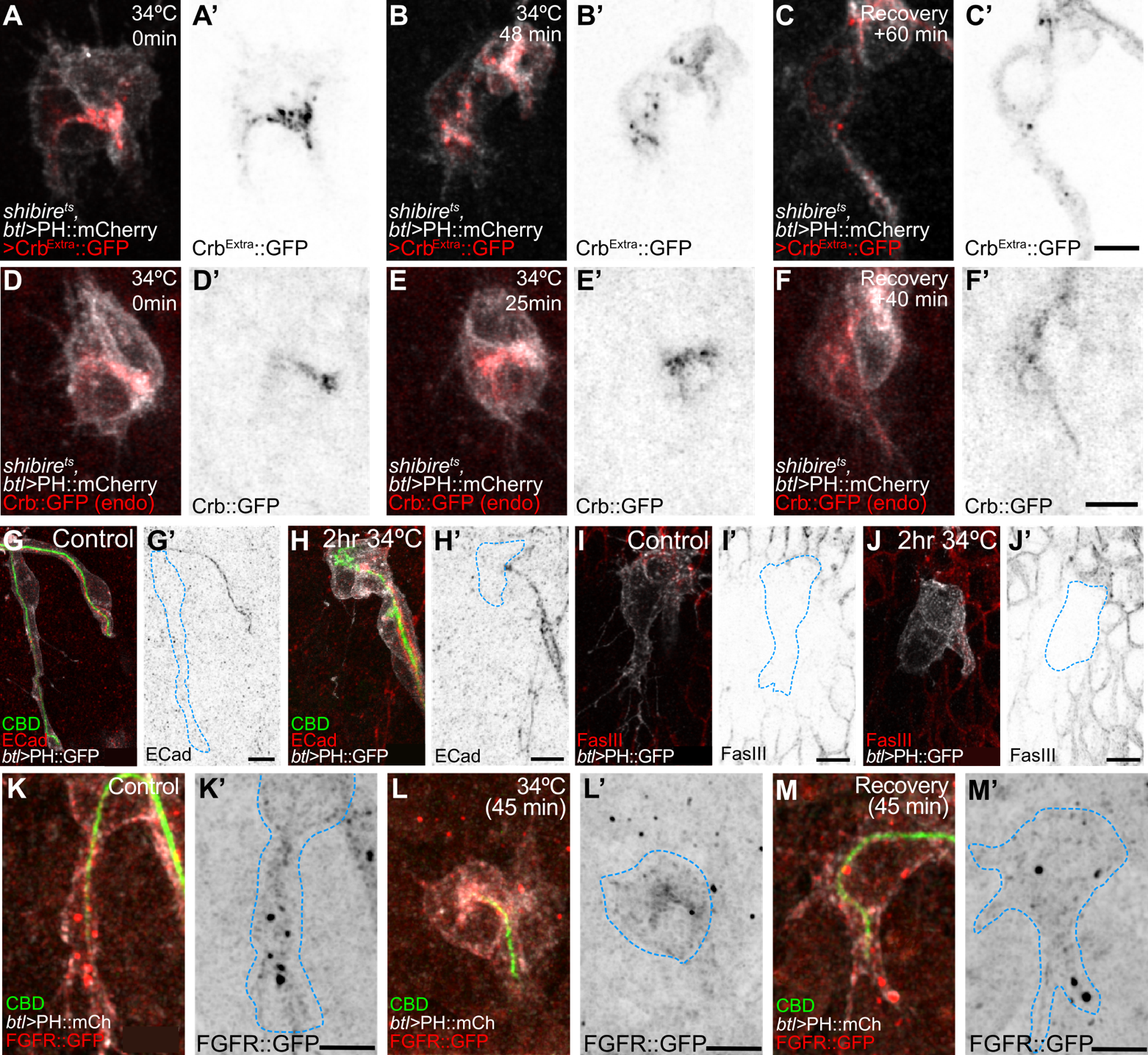


Terminal cells of *shibire^ts^* mutant embryos expressing PH::mCherry (A-F, K-M) or PH::GFP (G-J). Cells before dynamin inactivation (A, D, G, I, K), after (B, E, H, J, L) and at the end of recovery (C, F, M). (A-C) Distribution of the extracellular domain of Crb fused to GFP (Crb^Extra^::GFP). (D-F) Distribution of endogenously tagged Crb. (G-H) Distribution of E-Cadherin and a Chitin-Binding domain fused to Alexa-647 (CBD). (I-J) Distribution of Fasciclin III (FasIII). (K-M) Distribution of FGFR::GFP in stained embryos. Scale bars: 5μm
