# Supplementary material for: Transcytosis via the late endocytic pathway as a cell morphogenetic mechanism
Source: EMBO J. 2020 Jul 13;39(16):e105332. doi: 10.15252/embj.2020105332 (PMC7429744; doi:10.15252/embj.2020105332)
Supplement: Supplementary file 2 — Expanded View Figures PDF [file EMBJ-39-e105332-s002.pdf]

Expanded View Figures

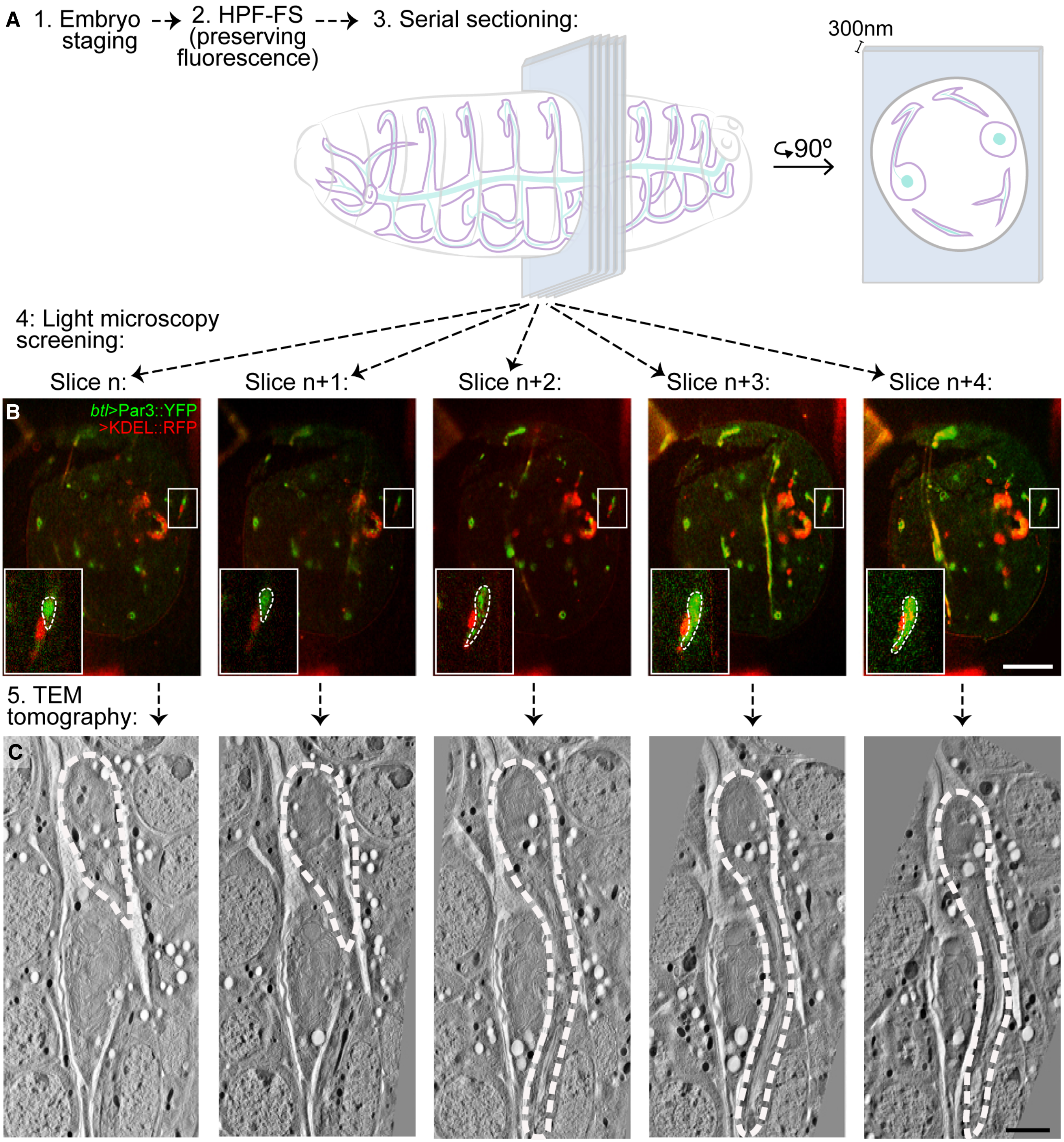

Figure EV1.

© 2020 The Authors

Figure EV1. Correlative light and electron microscopy workflow to identify terminal cells.

A Embryos were processed for EM while preserving the fluorescence signal, and then sectioned at 300 nm.
B Physical sections (slices) were then analysed by fluorescence microscopy, and once a terminal cell was identified (Slice *n*), the adjacent sections were collected to recover the complete cell (Slice *n* to *n*+4).
C The recovered slices were then imaged by electron tomography and digitally aligned.

Data information: Scale bars: 20 μm (B); 2 μm (C). White dashed line in (B and C) shows the outline of the terminal cell.

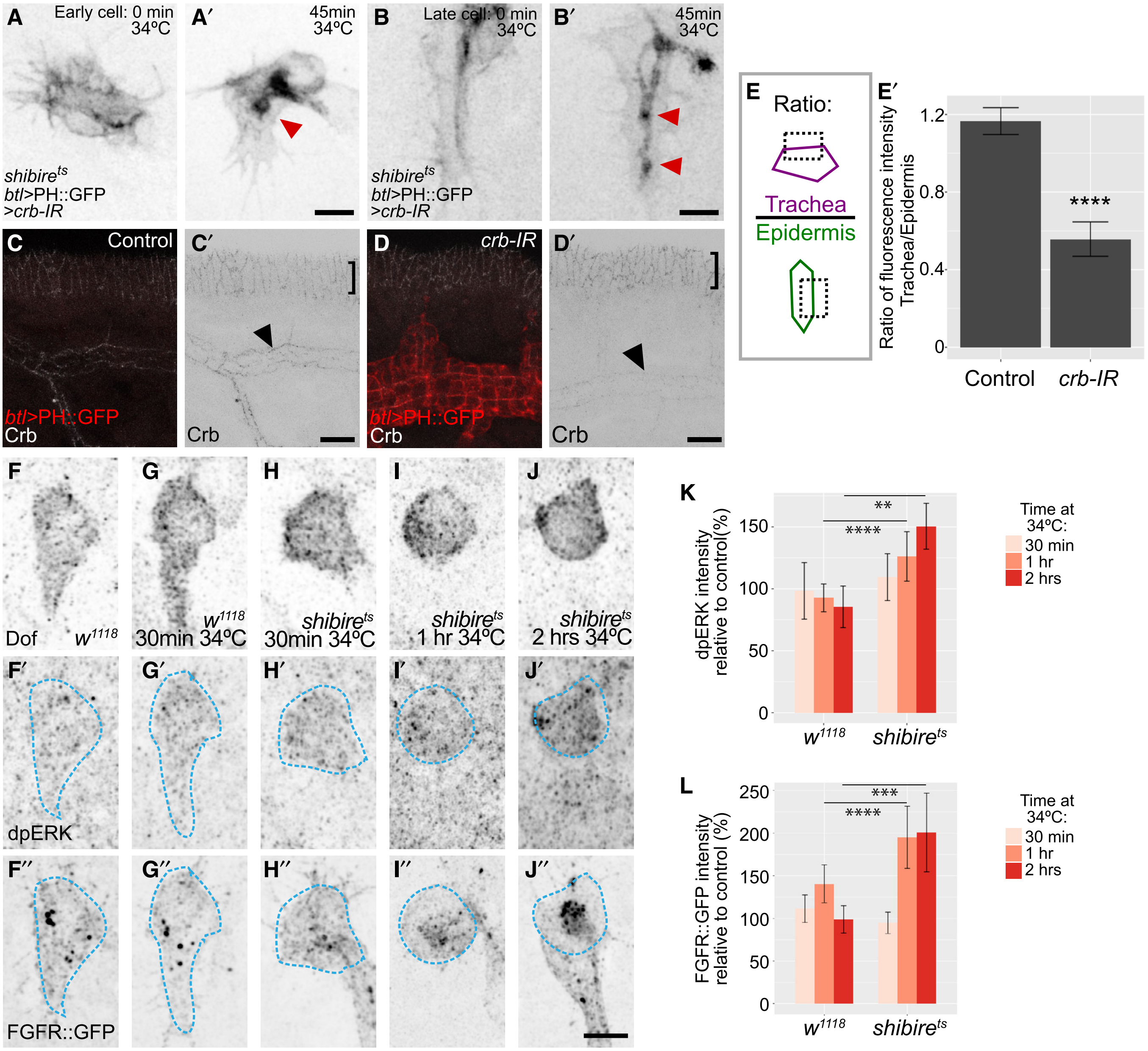

Figure EV2.

© 2020 The Authors

Figure EV2. Effect of blocking endocytosis on Crb and FGF signalling.

A, B Effect of Crb interference RNA (Crb-IR) on *shibire*^{ts} terminal cells. Red arrowheads point to sites of membrane accumulation in the apical compartment. (A) Cell at the onset of tube formation; (A) at the beginning of dynamin inactivation and (A′) after 45 min. (B) Later-stage terminal cell; (B) at the beginning of dynamin inactivation and (B′) after 45 min.

C, D Fixed embryos stained for Crb. Black arrowheads point to signal in the trachea and squared brackets show signal in the epidermis, which was used for normalization. (C) Control embryos (siblings without driver or UAS constructs). (D) Embryos expressing PH::GFP and *crb-IR* under *btl-gal4*.

E–E′ Quantification of Crb fluorescence intensity for 4 control and 5 *crb-IR* embryos. Data are plotted as ratio of signal in tracheal cells compared to epidermal cells, ± SD; ****$P > 0.0001$, two-tailed *t* test.

F–J Fixed embryos stained for Dof as a terminal cell marker, dpERK (F′-J′, terminal cell position is highlighted in blue) and FGFR::GFP from the fTRG collection (F″-J″).

K–L Quantification of dpERK and FGFR::GFP fluorescence intensity. Data are plotted as per cent relative to control (w^{1118}, for each time point), ± SD. We analysed two cells per embryo; number of embryos analysed: *w*, control (for 30 min at 34°C), $n = 13$; *w*, 30 min at 34°C, $n = 7$; *shi*, control (for 30 min at 34°C), $n = 2$; *shi*, 30 min at 34°C, $n = 11$; *w*, control (for 1 h at 34°C), $n = 18$; *w*, 1 h at 34°C, $n = 11$; *shi*, control (for 1 hr at 34°C), $n = 4$; *shi*, 1 h at 34°C, $n = 9$, *w*, 2 h at 34°C k, $n = 5$; *shi*, control (for 2 h at 34°C), $n = 6$; *shi*, 2 h at 34°C, $n = 6$. **$P = 0.009$, ***$P = 0.0002$, ****$P > 0.0001$, Kruskal–Wallis test and Dunn's test for multiple comparisons. Data information: Scale bars: 5 μm.

Source data are available online for this figure.

© 2020 The Authors

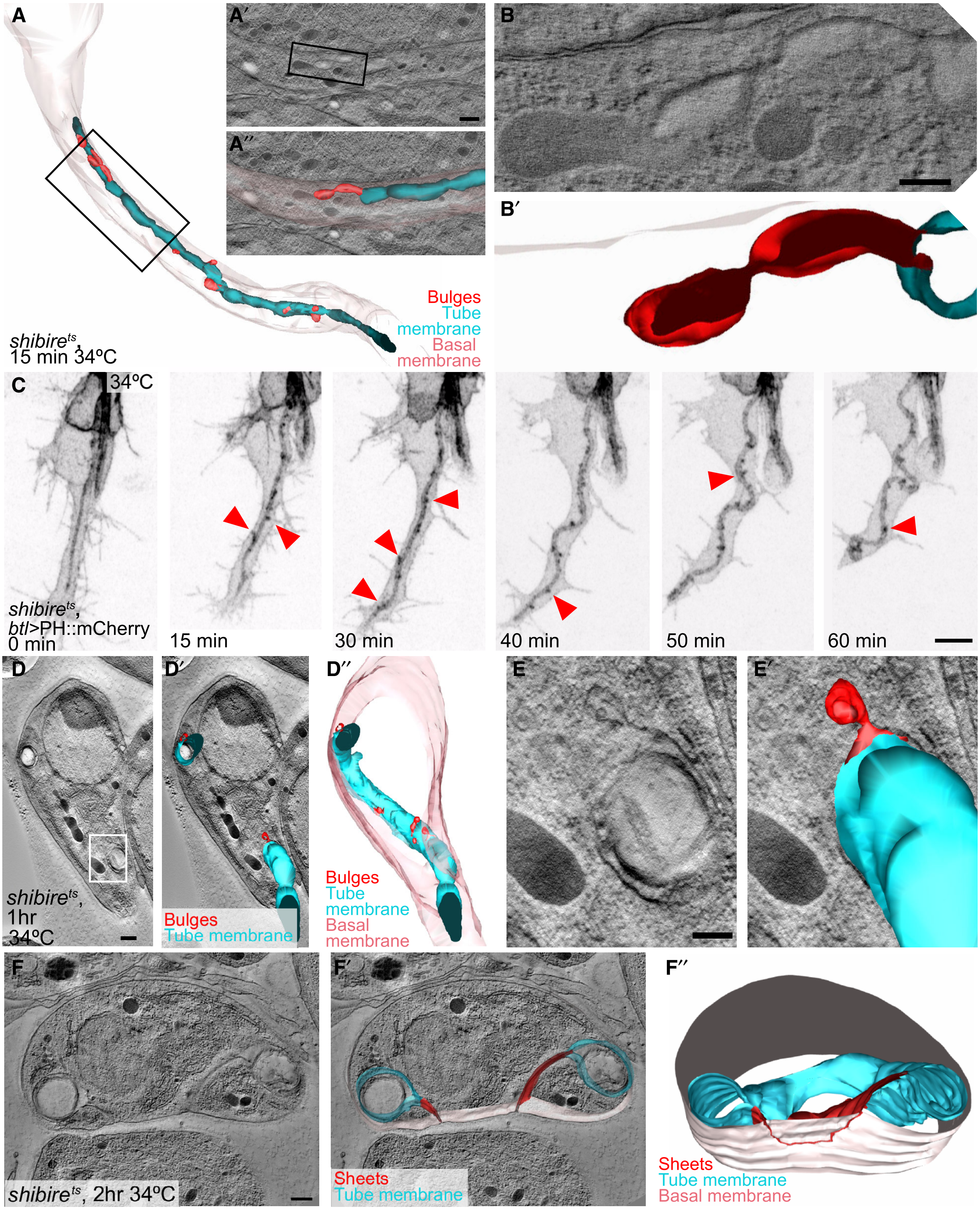
A
A′
A′′
B
B′
Bulges
Tube
membrane
Basal
membrane
shibire^ts,
15 min 34°C
C
34°C
shibire^ts,
btl>PH::mCherry
0 min
15 min
30 min
40 min
50 min
60 min
D
D′
D′′
E
E′
shibire^ts,
1hr
34°C
Bulges
Tube membrane
Bulges
Tube
membrane
Basal
membrane
F
F′
F′′
shibire^ts, 2hr 34°C
Sheets
Tube membrane
Sheets
Tube membrane
Basal membrane

Figure EV3.

Figure EV3. Effects of dynamin inactivation on membrane morphology.

A, B Older *shibire*^{ts} terminal cell that had already formed a long branch and tube before dynamin inactivation, fixed 15 min after inactivation. (A) Reconstruction of the entire cell. (A′, A″, B, B′) Higher magnification details of the cell, tomograms and reconstructions.

C *shibire*^{ts} terminal cell expressing PH::mCherry. Red arrowheads point to puncta of fluorescent material at the tube membrane.

D–F TEM tomograms and 3D reconstructions of older *shibire*^{ts} terminal cells similar to (A), but after 1 h (D–E) and 2 h (F) at restrictive temperature. Box in (D) is magnified in (E). (F″) The position at which the sheet between apical and basal is connected to the basal membrane is traced in red on the outside view of the basal membrane. The cells shown in (D–F) were found and acquired without the CLEM approach.

Data information: Scale bars: 500 nm (A, D, F), 200 nm (B, E), 5 µm (C).

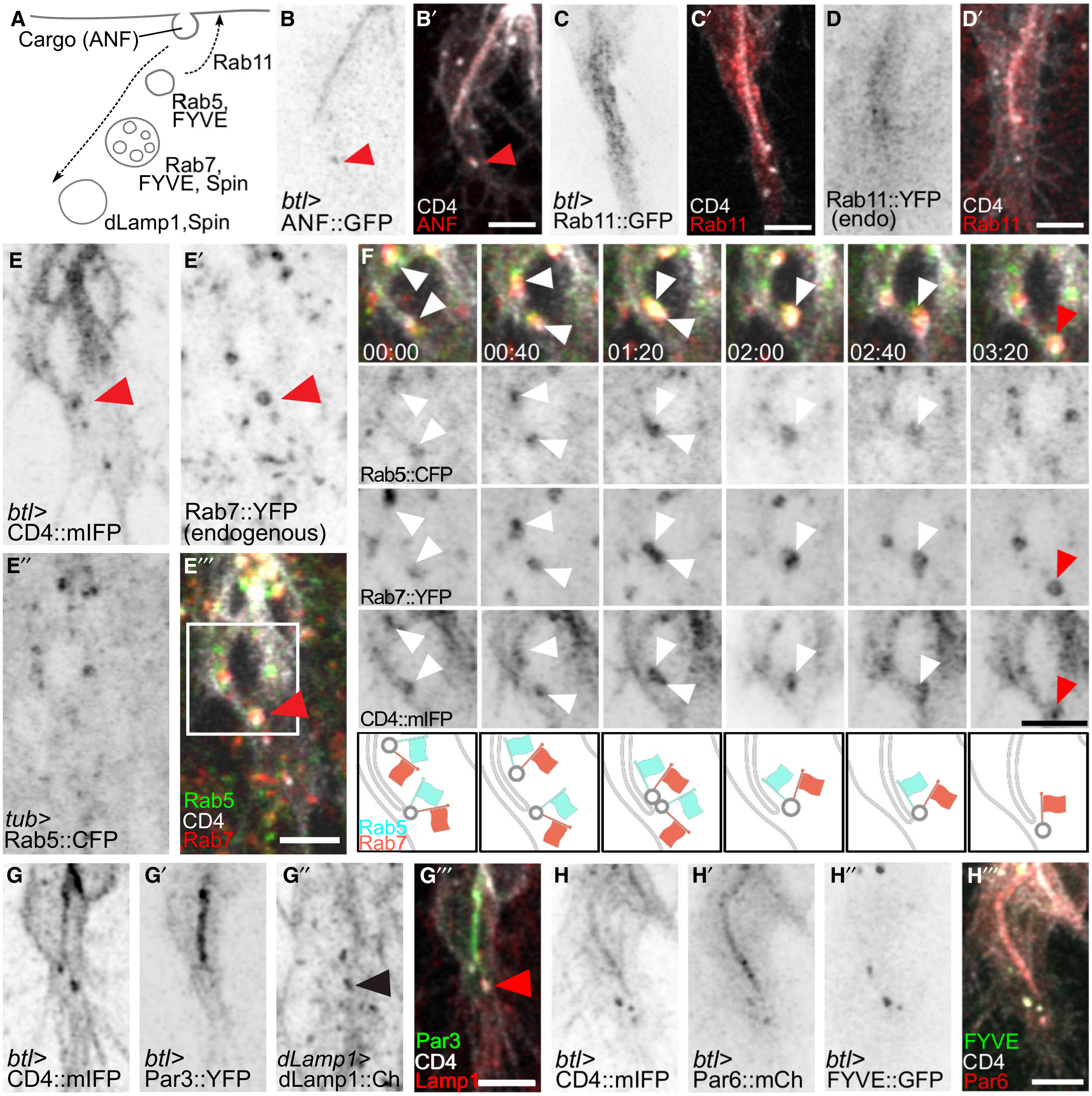

Figure EV4.

 © 2020 The Authors

Figure EV4. Distribution of late endosomal markers during terminal cell growth.

A–D Terminal cells expressing CD4::mIFP under *btl-gal4*, together with markers of different compartments (summarized in A): (B) ANF::GFP; red arrowheads: a CD4::mIFP vesicle that carries ANF::GFP; (C) Rab11::GFP under *btl-gal4*, (D) Endogenously YFP-tagged Rab11.

E, F Cell expressing CD4::mIFP under *btl-gal4*, Rab5::CFP under direct control of the tubulin promoter and endogenously tagged Rab7::YFP. (F) shows the area marked by the box in (E‴) at higher magnification and at six time points. White arrowheads: Rab5-positive, Rab7-positive CD4::mIFP vesicles; red arrowhead: Rab5-negative, Rab7-positive CD4::mIFP vesicle. A diagrammatic interpretation of the experiment shown in (E-E‴) is shown below.

G, H Terminal cells expressing CD4 together with (G) Par3::YFP and dLamp1::mCherry under its own promoter and (H) with Par6::mCherry and FYVE::GFP. Black and red arrowheads point to dLamp1::mCherry in a CD4 vesicle.

Data information: Scale bars: 5 μm.

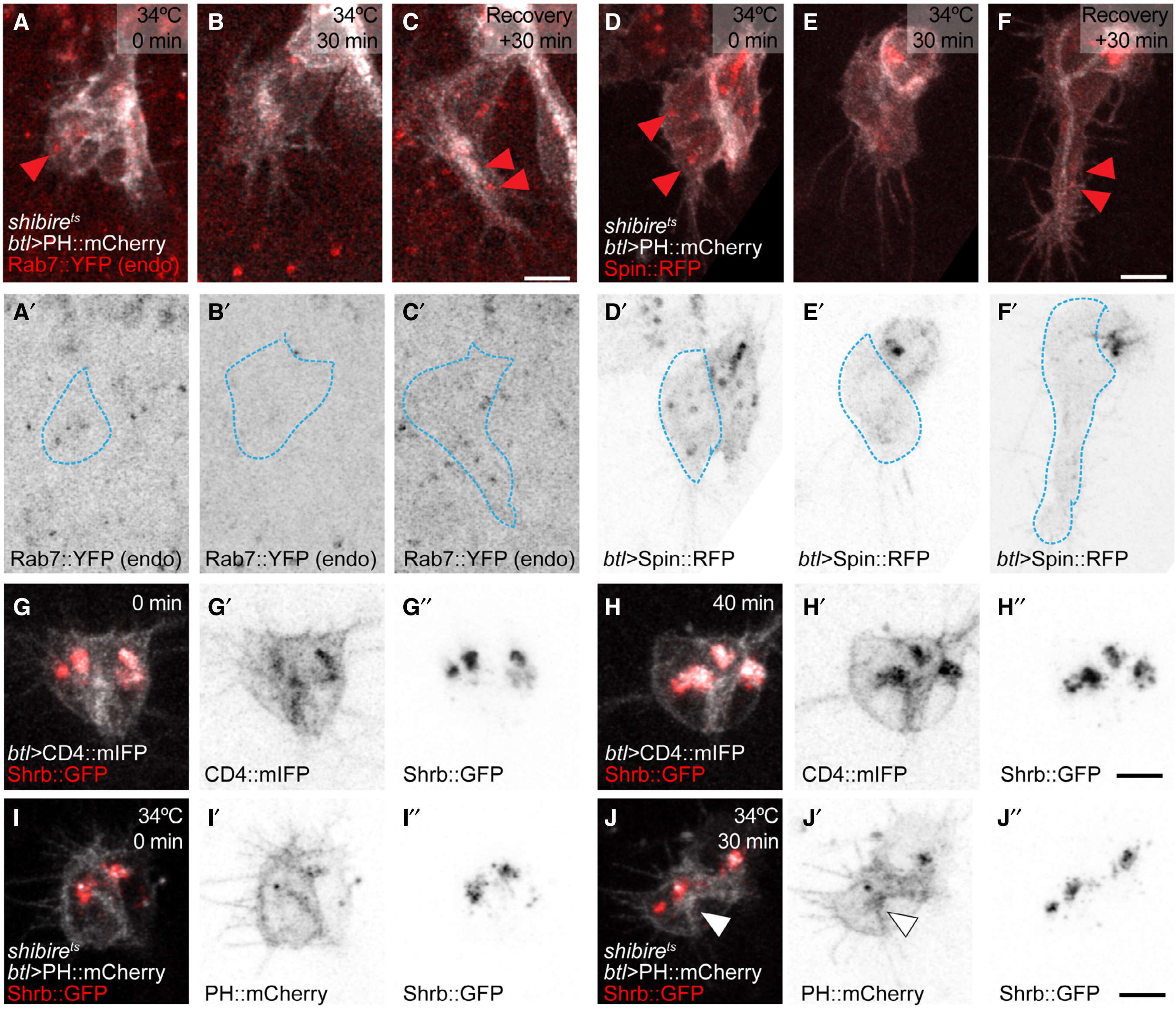

Figure EV5. Effect on dynamin inactivation on vesicles carrying late endocytic markers.

A–F *shibire^{ts}* cells expressing PH::mCherry under *btl-gal4*, together with endogenously labelled Rab7::YFP (A-C) or with Spin::RFP (D-F). The outlines of the terminal cells were traced using the mCherry fluorescence and superimposed on the image of the Rab7::YFP channel (blue broken line) to distinguish the cell from the surrounding tissue, which also expresses Rab7::YFP. Arrowheads point to Rab7 and Spin::RFP vesicles in the terminal cell.

G, H Terminal cell expressing Shrb::GFP and CD4::mIFP under *btl-gal4*, at the onset of tube formation (G) and 40 min later (H).

I, J *shibire^{ts}* terminal cell expressing Shrb::GFP and PH::mCherry under *btl-gal4* before dynamin inactivation (I) and after 30 min of inactivation (J). Arrowheads point to PH::mCherry accumulation in the apical compartment.

Data information: Scale bars: 5 µm.

 © 2020 The Authors
